# Summary Document Research on RDS Anti-addiction Modeling: Annotated Bibliography

**Published:** 2024-04-05

**Authors:** Kenneth Blum, David Baron, Thomas McLaughlin, Panayotis K. Thanos, Catherine Dennen, Mauro Ceccanti, Eric R. Braverman, Alireza Sharafshah, Kai-Uwe Lewandrowski, John Giordano, Rajendra D. Badgaiyan

**Affiliations:** 1 Center for Sports and Mental Health, Western University Health Sciences, Pomona, CA, USA; 2 The Kenneth Blum Behavioral and Neurogenetic Institute, LLC., Austin, TX, USA; 3 Behavioral Neuropharmacology and Neuroimaging Laboratory on Addictions, Clinical Research Institute on Addictions, Department of Pharmacology and Toxicology, Jacobs School of Medicine and Biosciences, State University of New York, Buffalo, NY, USA; 4 Department of Family Medicine, Jefferson University School of Medicine, Philadelphia, PA, USA; 5 Department of Translational and Precision Medicine, Sapienza University, Rome, Italy; 6 Division of Genetics, Department of Cell and Molecular Biology and Microbiology, School of Science and Biotechnology, University of Isfahan, Isfahan, Iran; 7 Department of Orthopaedics, Fundación Universitaria Sanitas Bogotá D.C. Colombia; 8 Division of Recovery Science, JC’S Recovery Center, Hollywood, Florida, USA; 9 Department of Psychiatry, Case Western Reserve University, School of Medicine, Cleveland, OH, USA

**Keywords:** Genetic addiction risk severity, Anti-addiction modeling, Substance use disorder

## Abstract

Annotated bibliography of genetic addiction risk severity (GARS) publications, pro-dopamine regulation in nutraceuticals (KB220 nutraceutical variants), and policy documents. Further research is required to encourage the field to consider “Reward Deficiency Syndrome (RDS) Anti-addiction Modeling” which involves early risk identification by means of genetic assessment similar to GARS, followed by induction of dopamine homeostasis by means of genetically guided pro-dopamine regulation similar to KB220. These results suggest that genetically based treatments may be a missing piece in the treatment of substance use disorder (SUD).

## Introduction

According to McLellan et al. [1], despite the enormous efforts made by the federal government to assist in funding, developing, and delivering certain treatments (Medication Assisted Treatment (MAT)) to people with SUD, the treatment penetration rate is less than 20%, despite the fact that there is no magic bullet or “cure” for SUD. They correctly point out that in the diabetes field facing a similar dilemma, increased treatment penetration through early-stage diabetes detection, referred to as “prediabetic.” In 2001, the American Diabetes Association suggested that the term prediabetic could be operationally defined by augmented scores on two laboratory tests: impaired glucose tolerance and impaired fasting glucose. Using this strategy led to an extensive campaign, partnership with third party payors, which has resulted in increased risk detection rates, shorter delays between symptom onset and treatment entry, and successfully halting progression to diabetes [2].

It is being advocated that “pre-addiction” be included in the DSM by Volkow (director of NIDA) and Koob (director of NIAAA). This proposal includes the possibility of developing a test for categorizing mild, moderate, and high risks for future addictive behavior. Based on our initial work and the work of many other global scientists, we have concluded that the pre-addiction classification is best described by a construct known as dopamine dysregulation at the mesolimbic reward circuit, specifically reward deficiency or net hypo-dopaminergia. A total of 1,457 articles are listed in PubMed, of which approximately 47% are independent of our lab. A total of 224 articles are listed using the search term “Reward Deficiency Syndrome”. Although the term pre-addiction resonates well with the historical advances in the diabetic field, scientifically the real evidence rests on concepts related to brain neurotransmitter deficits, or even surfeit (especially during adolescence as a neurodevelopmental event) referred to as reward dysregulation. In the DSM-V, the DSM-V defines SUDs along a 3-stage severity continuum based on 11 equally weighted symptoms of impaired control. There are approximately 4% to 5% of adults who suffer from severe SUD, defined by 6 or more symptoms. In contrast to those with severe SUD (i.e., addiction), those with severe SUD (two to five symptoms) make up a much smaller proportion of the adult population (13%) and are thus responsible for a greater proportion of substance use-related harms to society. Treatment efforts and public health policies have largely ignored individuals with early-stage SUDs, instead focusing almost exclusively on those with serious, chronic addictions. In mainstream health care settings, where it is most common, very little has been done to identify and monitor harmful substance misuse and early-stage SUDs. Early-stage SUD has no commonly understood name among clinicians or the general public. In this regard, we are proposing “Reward Deficiency” (meaning lack of normal function) or “Reward Dysregulation” as a general term which does encompass the nosology of “pre-addiction” as well. Having made this suggestion, we are aware that the latter terminology would be more understandable for the public. The latter seems more reasonable to the DSM and psychiatrists and other clinicians.

Regardless of the appropriate name, in a similar vein to the concept of “prediabetes,” the development of a reliable system for early identification of people at risk for substance abuse and other behavioral addictions (pre-addiction) is important. In order to capture the psychological correlates of RDS, we propose the GARS [3] test as well as the RDSQ2920 pencil and paper test. PubMed lists 58 articles regarding GARS, despite requiring additional research. It’s unfortunate that most of the results are from Blum’s laboratory and the content is mostly narrative, but they are still encouraging.

Several studies have shown that objective DNA polymorphic identification can be used to identify drug and alcohol risks in more effective ways than just subjective (but still useful) diagnostic surveys such as family history [4]. The field of clinical research has published many important works in a variety of areas. The DNA guided pro-dopamine regulation (KB220) is also the subject of a number of clinical trials to resolve the RDS dilemma.

In addition to addictions and compulsive and impulsive behaviors, RDS encompasses a wide range of mental health disorders. This disorder is described as an octopus of behavioral dysfunction [5], resulting from genetic and epigenetic influences that break down the cascade of rewards. Physiological drives that result in reward neurotransmission defects interfere with the pleasure derived from satisfying those drives. Clinical trials and animal imaging have demonstrated pro-dopamine regulatory function of formulations of KB220, a nutraceutical. Depressive and schizophrenic cohorts show significant overlap in dopaminergic gene polymorphism allele risk [6], according to large Genome-Wide Association Studies (GWAS) studies. Additionally, attention deficit hyperactivity disorder (ADHD), post-traumatic stress disorder (PTSD), and spectrum disorders have been linked to RDS [7] neurogenetic and psychological underpinnings.

Behavior disorders are endophenotypes of RDS, the true phenotype. Would it be logical to wonder if RDS exists everywhere? With a multitude of neurotransmitters and polymorphic loci influencing behavioral functionality, RDS could be regarded as foundational in species evolution and survival.

With this mind and in helping to fulfill the necessary requirements as suggested by the construct of “pre-addiction” [1] and possible assessment of early identification [8–12] and trichotimization of mild, moderate and high predictive risk for RDS behaviors including SUD, with a potential solution involving DNA directed pro-dopamine regulation and subsequent induction of dopamine homeostasis, the following annotated bibliography selected from our laboratory only, having large support from many published articles by others over the last 3 decades, may serve a heuristic purpose.

## Annotated Bibliography

### GARS

#### Allelic association of human dopamine D2 receptor (DRD2) gene in alcoholism [13]

An allelic association between alcoholism and the DRD2 gene has been discovered in a blind experiment. A DNA fragment containing the entire 3’ coding exon, the polyadenylation signal, and approximately 16.4 kilobases of the noncoding 3’ sequence of the human DRD2 gene (lambda hD2G1) was probed with restriction endonucleases from 70 brain samples taken from alcoholics and non-alcoholics. In these samples, 77% of alcoholics were correctly classified as alcoholics by A1 allele, while 72% of non-alcoholics were correctly classified as nonalcoholic. A gene located on chromosome 11 q22-q23 may confer susceptibility to alcoholism at least in part.

#### Alcoholism and the DRD2 gene [14]

A review by Noble and Blum [14] examined the association between the DRD2 locus Al allelic variation and alcoholism. According to them, there is no association. The data presented by Noble and Blum [14] and other analyses relevant to this association have been reanalyzed in response to this assertion. Our findings are as follows: 1. According to Noble and Blum [14], the Al frequency for unscreened controls is 0.19 (n = 307), while for screened (alcoholics excluded) controls is 0.11 (n = 186). Between the two controls, a significant difference is observed (κ^2^,5.17; p = 0.02). To compare alcoholics with controls, it is vital that carefully screened controls be used. According to Noble and Blum [14], the Al frequency in the alcoholics (severe and not severe) is 0.19 (n = 502) when compared with the screened controls.

#### Genetic predisposition in alcoholism: association of the DRD2 TaqI B1 RFLP with severe alcoholics [15]

DRD2 gene allele 3’ Taq1 A1 has been associated with severe alcoholism in previous studies. An associational study of the 5’ Taq1 B alleles with alcoholism and its comparison with the 3’ Taq1 A alleles has been conducted recently as a result of a new polymorphism discovered closer to the regulatory regions of this gene. We analyzed 133 blood samples of non-alcoholics, less severe alcoholics, and severe alcoholics using restriction fragment length polymorphism methodology. White subjects (n = 115) with and without alcoholism do not show any significant difference in the prevalence of the B1 allele. In contrast, severe alcoholics (n = 49) have a significantly higher prevalence of this allele compared to non-alcoholics (p = 0.008) and less severe alcoholics (p = 0.005). Only in the severe alcoholic group is the prevalence of Taq1 A alleles significantly higher than Taq1 B alleles in whites carrying the Taq1 A allele. Accordingly, severe alcoholism can be caused by both alleles of the DRD2 gene at the 5’ and 3’ ends. There might be an etiological role for the DRD2 gene in some severe alcoholics, based on this.

#### Association of the A1 allele of the DRD2 gene with severe alcoholism [16]

Based on peripheral lymphocyte DNA as the source of DNA, 159 subjects, including non-alcoholics (n = 43), less severe alcoholics (n = 44), severe alcoholics (n = 52) and young children of alcoholics (n = 20), were examined to determine their allelic association with the DRD2 gene. A significant association was found between the combined alcoholic group and the non-alcoholic group for the A1 allele of DRD2 gene. Moreover, severe alcoholics and non-alcoholics showed an even stronger association. In addition, non-alcoholics and alcoholics showed significant differences in their A1 allele associations. There are two independent components to the risk of alcoholism severity: family history and having the A1 allele. Race was also taken into account when analyzing DRD2 genotypes and allelic frequencies. In the total sample of blacks, the A1/A1 genotype was more common than in the total sample of whites, and the frequency of A1 alleles was higher than in the total sample of whites. In addition, both blacks and whites with severe alcoholism had a greater frequency of the A1 allele than non-alcoholics. This racial difference needs to be further investigated, however, because there is only a small sample size of blacks. An association between severe alcoholism and the A1 allele of the DRD2 gene is strongly supported by this study of the largest sample of alcoholics to date.

#### Allelic association of the DRD2 gene with receptor-binding characteristics in alcoholism [17]

A study of 66 brains of alcoholics and non-alcoholics determined the allelic association between the human DRD2 gene and its binding characteristics. A 1.5-kilobase digest of a clone of the human DRD2 gene (lambda hD2G1) was probed with DNA from the cerebral cortex treated with Taq A1. The binding characteristics (Kd (binding affinity) and Bmax (number of binding sites)) of the DR2 were determined in the caudate nuclei of these brains using tritiated spiperone as the ligand. A significant difference was observed between alcoholics and non-alcoholics in the adjusted Kd. Bmax was significantly reduced in subjects with the A1 allele who had a high association with alcoholism, as compared to subjects with the A2 allele. Furthermore, subjects with A2/A2, A1/A2, and A1/A1 alleles had progressively reduced Bmax values, with subjects with A2/A2 having the highest mean values, and subjects with A1/A1 having the lowest. At least one subtype of severe alcoholism appears to be influenced by the polymorphic pattern of the DRD2 gene and the differential expression of its receptors.

#### Allelic association of the DRD2 gene with cocaine dependence [18]

DRD2 allele prevalence was examined in cocaine-dependent (CD) Caucasian (non-Hispanic) male subjects, and the relationship between DRD2 allele prevalence and family history and selected behavioral measures was examined. CD subjects (n = 53) possessed the A1 allele at a prevalence of 50.9%. There was a significantly greater prevalence in CD subjects (n = 53) than in non-substance abuse controls (n = 100) and in population controls (n = 265) that did not exclude substance abusers. Similar to CD subjects, non-substance abuse controls (n = 53) showed a significantly higher prevalence (p = 10^−2^) of the B1 allele. The A1 allele was significantly associated with potent routes of cocaine use and the interaction between early deviant behaviors and parental alcoholism in CD subjects. As a result of the cumulative number of these three risk factors, A1 allelic prevalence was positively and significantly correlated with the cumulative number of these three risk factors in CD subjects (p > 10^−3^). A gene on chromosome 11’s q22-q23 region confers susceptibility to this drug disorder based on the results of studies that strongly associate the minor alleles (A1 and B1) of DRD2 with CD.

#### Can genetic testing coupled with enhanced dopaminergic activation reduce recidivism rates in the workers compensation legacy cases? [19]

There are genetic variations in the world population that increase the risk of substance abuse or behavioral addictions (e.g., gambling, internet gaming, multiple sexual partners, etc.). Involuntary indulgence in detrimental and destructive behaviors may prevent them from reaching their optimum health potential, contribute to impaired health, or prevent them from reaching their optimum health potential. Genetic reward deficiency can lead not only to compulsions and excessive cravings but also to poor decision making. Eventually, this brain hard wiring will result in narcotic addiction; antisocial behavior; crime and unnecessary medical procedures. Using a paradigm shift in both genetic testing and improving functional connectivity of the brain with pro-dopamine regulation (KB220) should result in a better quality of life for “Legacy Claim” patients since the major underlying root causes of opiate dependency will be reduced. As a result, workers’ compensation abuse will be curtailed, and employers and carriers will save money each month. Our approach has successfully treated a hypo-dopaminergic genetically tested patient with GARS who had a workers compensation payment for medications over $50,000 per month for over two years, with KB220 as well as other natural therapies. The main goal of our program is to “redeem joy” while inducing higher functionality that leads to returning to work. It is recommended to interpret cautiously after additional research has been completed and further confirmation has been obtained.

#### Coupling GARS with electrotherapy: Fighting iatrogenic opioid dependence [20]

Prescription drug abuse induced by legal opioids is a major concern worldwide. Dopaminergic tone plays a significant role in the neurophysiology of pain relief, which provides potential therapeutic solutions. Within the last 30 days, approximately 8.7% of the United Stats (US) population over the age of 12 had used a psychoactive drug. According to one study, genetics contributed approximately 60% to the variance of SUD, but candidate genes evaluated by GWAS had relatively small effects. The purpose of this paper is to propose a number of alternative strategies for combating this global endemic. A strategy that targets (1) high-dosage medical users; (2) those seeking care from multiple doctors; (3) those involved in drug diversion; (4) genetic testing to determine addiction liability and severity indices; and (5) non-pharmacological analgesic treatments such as electrotherapy should be employed to prevent death due to opioid overdose and attenuation of prescription abuse.

#### Frequency of the dopamine receptor D3 (DRD3) (rs6280) vs opioid receptor μ1 (rs1799971) polymorphic risk alleles in patients with opioid use disorder (OUD): A preponderance of dopaminergic mechanisms? [21]

In spite of opioids’ power to inhibit pain signals, the current epidemic of OUD and overdose deaths has tarnished their use. There are still gaps in our understanding of opioid receptor mechanisms and their role in opioid seeking behavior, despite published reports. Molecular, neurogenetic, and neuropharmacological insights are therefore needed to understand OUD. Rather than being exclusively related to a particular drug of choice, an addictive endophenotype may be generalizable to altered brain reward circuits affecting net mesocorticolimbic dopamine release. The self-administration of opioids and other drugs may result from genetic or epigenetic alterations across the dopaminergic reward system. Knocking out the DRD3, for instance, increases opioid vulnerability. Using a sophisticated polymorphic risk analysis in a human cohort of chronic opioid users, we found that DRD3 (rs6280) has a higher frequency than opioid receptor 1 (rs1799971). While opioidergic mechanisms may contribute to OUD, African Americans may view opioid-seeking behavior primarily through dopamine-related receptors. A focus on the regulation of dopaminergic homeostasis by DRD3 may be required for novel and improved neuropharmacological therapies targeting OUD.

#### Insurance companies fighting the peer review empire without any validity: The case for addiction and pain modalities in the face of an American drug epidemic [22]

There is a rising opioid overdose epidemic in the US; we are challenged to offer non-addictive/non-pharmacological pain relief alternatives. Chronic pain can be effectively managed without opioids using proven strategies. Peer-reviewed scientific studies warn against chronic opioid use, as well as the lack of evidence to support long-term opioid use for pain, but utilization review providers for insurance companies ignore these studies. Changing American chronic pain management’s drug-embracing culture requires a paradigm shift. Pushbacks from insurance companies can be a barrier to treatment, particularly when it comes to battling against pain relief alternatives. In the US, pain specialists must find alternatives to analgesics that do not cause tolerance and subsequent biological induction of the “addictive brain.” It is worth noting that the reward center of the brain plays a vital role in modulating nociception, and that changes in dopaminergic circuitry may affect several sensory and affective components of chronic pain syndromes. It may be possible to eliminate guesswork when it comes to addiction by knowing a patient’s GARS.

#### Proposing a “brain health checkup (BHC)” as a global potential “standard of care” to overcome reward dysregulation in primary care medicine: Coupling genetic risk testing and induction of “dopamine homeostasis” [12]

Opioid overdoses contributed to more than 100,000 premature deaths in 2021. Genetic antecedents and epigenetic insults contribute to reward dysregulation with neuropsychiatric and cognitive impairments. An extensive meta-analysis involving millions of individuals found frequent comorbidity between depression and SUD. In addition to NEGR1, DRD2 in the nucleus accumbens (NAc) were found to be associated with significant associations. In spite of the rise in substance abuse disorders and neuropsychiatric illnesses, there are no routine objective brain assessments. In order to treat clinical syndromes in psychiatric patients, a standard objective BHC is encouraged. In addition to memory, attention, neuropsychiatry, and neurological imaging, the BHC would consist of reliable, accurate, cost-effective, objective assessments. Utilizing primarily PubMed, over 36 years of virtually all the computerized and written-based assessments of memory, attention, psychiatric, and neurological imaging were reviewed, and the following assessments are recommended for use in the BHC: Central Nervous System Vital Signs (Memory), Test of Variables of Attention (Attention), Millon Clinical Multiaxial Inventory III (Neuropsychiatric), and Quantitative Electroencephalogram (qEEG)/P300/Evoked Potential (Neurological Imaging). Finally, we recommend continuing research into incorporating a new standard BHC coupled with qEEG/P300/Evoked Potentials and genetically guided precision induction of “dopamine homeostasis” to diagnose and treat reward dysregulation to prevent epigenetically passing on the consequences of dopamine dysregulation to future generations.

#### Americas’ opioid/psychostimulant epidemic would benefit from general population early identification of genetic addiction risk especially in children of alcoholics [23]

A system for identifying and monitoring at-risk adolescents’ drug use/abuse would have substantial implications for public health, as well as better understanding addiction’s underlying biology. Defeating an epidemic of this magnitude and size will require bold, innovative interventions and screenings, scientifically based educational campaigns, and DNA data protection measures. Despite the fact that one can opt out of these programs, the authors present a potential strategy to achieve these important objectives.

#### Neurogenetic impairments of brain reward circuitry links to RDS: Potential nutrigenomic induced dopaminergic activation [24]

In both inpatient and outpatient facilities, our laboratory used the Comprehensive Analysis of Reported Drugs (CARD^™^) to identify a significant lack of compliance with prescribed treatment medications and a lack of abstinence from drugs of abuse during active recovery. As a result of this unpublished research, we are able to develop accurate genetic diagnosis and holistic approaches that will promote brain reward circuitry in the mesolimbic dopamine system in a safe and effective manner. Using genes associated with dopaminergic function as an example, this editorial discusses the neurogenetics of brain reward systems. In order to expand our understanding of SUD, process addictions, and obsessive, compulsive, and impulsive behaviors, the term “Reward Deficiency Syndrome” has been used to describe behaviors associated with gene-based hypodopaminergic function. Natural and unnatural rewards play a key role in motivating and reinforcing behavior in this editorial. Besides this, it briefly discusses how a panel of reward genes, referred to as the GARS, can be used in combination with natural DRD2 agonist therapy. Developed from fundamental genomic research, it serves as a springboard for combining novel approaches to prevention and treatment of RDS.

#### Neurobiology of KB220Z-glutaminergic-dopaminergic optimization complex (GDOC) as a liquid nano: Clinical activation of brain in a highly functional clinician improving focus, motivation and overall sensory input following chronic intake [25]

By utilizing neurogenetics and epigenetics in research and neuroimaging, we are unraveling the mysteries of brain function, particularly as it relates to RDS. Drugs or nutraceuticals that enhance dopamine homeostasis and reduce dopamine resistance are encouraged. KB220Z is a liquid (aqua) nano GDOC that promotes high performance under work-related stress. One month was taken by the subject on GDOC. A half-ounce of GDOC was administered twice daily by the subject self-administering. In the first three days, a unique brain activation occurred; resembling white noise after 30 min and then dissipating after 45 min. In addition to improving his sense of smell and sleep, he described the effects as if his eyesight had improved slightly. Dopamine may be responsible for the calming effect the subject experienced during meditation. Additionally, he reported a control of going over the edge after a hard day’s work, which was accompanied by an increase in energy, motivation to work, focus, and multitasking, as well as a clearer focus on the task at hand. As a result of his increased Behavior Activating System (reward) in a social setting, the subject reported feeling less inhibited in a social setting and suggested Syndrome that GDOC increased his Behavior Inhibition System (caution). According to these and other studies, mood, work-related focus, and sleep were improved. There may be direct or indirect dopaminergic interactions responsible for these subjective feelings of brain activation. However, we need to conduct more research in a larger randomized placebo-controlled study to explore the role of GDOC, especially in nano-sized products, in order to determine how it affects circuit inhibitory control and memory banks, and whether it induces dopamine homeostasis regardless of hypo- or hyper-dopaminergic traits.

#### Introducing precision addiction management (PAM) of RDS, the construct that underpins all addictive behaviors [26]

Thousands of people around the world struggle daily with their frustrating and sometimes fatal love affair with getting high; for others, feeling good may be considered “high.” In order to improve understanding of the complex functions of brain reward circuitry, which plays a key role in addiction symptomatology, the neuroscience community conducts and funds outstanding research using sophisticated neuroimaging and molecular-genetic technology. In spite of the widespread recognition that dopamine plays a major role in behavioral and substance addictions, there is still controversy regarding how to modulate dopamine clinically in order to treat and prevent them. Biphasic blockade followed by long-term upregulation may be a prudent approach. Reward deficiency and stress-like anti reward symptomatology of addiction would be targeted with treatment to enhance brain reward functional connectivity volume. The GARS^®^ can be used to characterize such phenotypes. Through PAM^®^, which customizes neuro-nutrient supplementation based on GARS test results, and behavioral interventions, dopamine homeostasis can be achieved.

#### Hypothesizing that neuropharmacological and neuroimaging studies of GDOC (KB220Z) are associated with “dopamine homeostasis” in RDS [27]

RDS is a disorder characterized by addictive behaviors, both substance-related and non-substance-related. In spite of the US Food and Drug Administrations (FDA) approval of pharmaceuticals under the heading of MAT, these drugs do not provide optimal benefits. According to our hypothesis, these drugs work by blocking dopamine function, causing psychological extinction. A long-term use of buprenorphine/naloxone (Bup/Nal), however, can result in unwanted addiction liability, reduced emotional affect, and depression, as well as suicidal thoughts. As a result, we propose a paradigm shift in addiction treatment with the long-term goal of achieving “dopamine homeostasis.” While this is a noble goal, it will be very challenging. This commentary briefly summarizes past history of developing and subsequently utilizing a GDOC (Kb220Z) shown to be beneficial in at least 20 human clinical trials and in a number of published and unpublished studies. The cited studies indicate enhanced functional connectivity, connectivity volume, and perhaps, neuroplasticity in resting states, although more studies are necessary to confirm these findings. We present a Reward Deficiency Solution System that includes GARS, CARD, and a GDOC (Kb220Z). Continued investigation of this novel strategy may lead to a better-targeted approach in the long-term, causing dopamine regulation by balancing the glutaminergic-dopaminergic pathways. This may potentially change the landscape of treating all addictions leading us to the promised land.

#### Precision behavioral management (PBM) and cognitive control as a potential therapeutic and prophylactic modality for RDS: Is there enough evidence? [8]

An overview of the available and relatively new precision management of reward deficiencies manifested as substance abuse and behavioral disorders is provided in this brief commentary. A substantial body of evidence supports the efficacy of this potential therapeutic and prophylactic treatment modality, including current and future advances, concepts, and evidence-based guidelines. Originally conceptualized as PAM, PBM (coupling GARS with KB220) certainly deserves consideration as a valuable treatment modality for people with neurobiologically expressed RDS that suffer from impaired cognitive control in reward processing.

#### Molecular genetic testing in RDS: Facts and fiction [28]

Dopamine is released within the brain as a result of the Brain Reward Cascade (BRC) which is an interaction between neurotransmitters and their respective genes. RDS was coined to define addictive behaviors and their genetic components, and any variation within this pathway may result in addictive behavior. In order to conduct this review, we searched several databases including Psych INFO, ACP PIER, Psych Sage, PubMed/Medline, and Cochrane Systematic Reviews. Among the major search terms were dopamine agonist therapy for addiction; dopamine agonist therapy for reward dependence; dopamine antagonistic therapy for addiction; and dopamine antagonistic therapy for reward dependence. RDS behavior has been linked to a genetic component in many studies, but not all of them are scientifically supported. In spite of our bias, this Clinical Pearl discusses the facts and fictions surrounding molecular genetic testing in RDS and the significance behind the development of the GARS (GARSPREDX^™^), the first test to accurately predict one’s genetic risk for this disorder.

#### Global opioid epidemic: Doomed to fail without genetically based precision addiction medicine (PAM^™^): Lessons learned from America [29]

Overdose deaths from heroin and fentanyl have soared globally for men and women of all ages, social status, and economic status. The U.S. has seen an alarming increase in the number of overdose deaths caused by narcotics since 2010. Fentanyl’s rise is primarily driven by drug dealers who sell it as heroin, lace cocaine with it, or make illegal counterfeit opioids from it. In fact, the US Center for Disease Control (CDC) released data showing that opioid overdoses were up 15% in the first three quarters of 2016 compared to 2015. According to the President’s Commission on the crisis, the death toll is the same as “September 11^th^ every three weeks.” Government agencies, including NIDA, are actively trying to find a solution. To induce “dopamine homeostasis,” however, the scientific community must embrace genetic addiction risk coupled with precision or personalized medicine. A ten-gene and eleven single nucleotide polymorphism (SNP) panel can now predict Addiction Severity Index (ASI) for alcohol and drugs of abuse (e.g., opioids). The GARS^™^ showed a significant correlation with the Addiction Severity Index-Media Version (ASI-MV) derived alcohol and other drug severity risk scores in a multi-addiction center study. In a number of neuroimaging studies, we also display that BOLD dopamine activation across the brain reward circuitry improved resting state functional connectivity as well as volume connectivity in both animal (bench) and abstinent Chinese severe heroin-dependent patients (bedside). Studies linking gene polymorphisms to altered KB220Z have also revealed improved clinical outcomes in obesity patients.

#### Analysis of evidence for the combination of pro-dopamine regulator (KB220PAM) and naltrexone to prevent OUD relapse [30]

Naloxone or narcotic antagonists (NTX) were first demonstrated to be effective in treating alcohol dependence in Blum’s laboratory. In conjunction with many other studies, this seminal work published in Nature in the early 1970’s led to the development of NTX now used for both alcohol and opioid dependence treatment. XR-NTX was approved by the FDA in 2006 as an extended-release injectable of naltrexone. Naltrexone is a relatively weak antagonist of κ- and δ-receptors and is also a potent μ-receptor antagonist. Dosages of naltrexone that effectively reduce opioid and alcohol consumption also actively block μ-receptors, but chronically down-regulate mesolimbic dopamine release. While studies show short-term benefits, ongoing evidence indicates that adherence to NTX is not high enough to qualify as adherence. Despite higher rates of concurrent non-opioid substance use, extended release NTX opioid treatment has superior outcomes, including less likely relapse (defined as daily use), and much longer time to relapse. There was higher compliance with long-term extended-release injectable (XR-NTX) for opioid dependence than for alcohol dependence. It is imperative to consider modalities in combination with XR-NTX. Blum et al. [30] study showed that a combination of naltrexone and KB220, a neuro-nutrient that regulates dopamine, significantly prevented opioid relapses. In addition to early identification of addiction vulnerability, genetically guided therapy with the KB220 neuro-nutrient referred to as PAM will provide valuable information regarding genetically guided therapy using polymorphic risk alleles from ten reward circuitry genes.

#### RDS surprisingly is evolutionary and found everywhere: Is it “blowin’ in the wind?” [31]

In addition to addictions and compulsive and impulsive behaviors, RDS encompasses a wide range of mental health disorders. An octopus of behavioral dysfunction, RDS is a genetic and epigenetic disorder that causes abnormal behavior due to a breakdown in neurotransmission. Physiological drives that satisfy powerful human needs are interfered with when reward neurotransmission deficiencies occur. In animal and human neuroimaging and clinical trials, KB220, a nutraceutical with prodopamine regulatory properties, may be useful for epigenetic repair using precision gene-guided therapy (GARS). Dopaminergic gene polymorphisms have recently been linked to depression and schizophrenia in large GWAS studies. There is also a large body of literature identifying ADHD, PTSD, and spectrum disorders as having neurogenetic and psychological underpinnings. It is hypothesized that behavioral disorders are endophenotypes of RDS, while the true phenotype is RDS. Do RDS exist everywhere? Is this logical? Although complex, RDS, with an array of neurotransmitters and polymorphic loci influencing behavioral function, may be crucial to species evolution and survival, rather than intangible.

#### GARS^™^ as a predictor of SUD: Identifying predisposition not diagnosis [32]

There is indeed a synergistic link between these polymorphisms and an overall expression of DNA predictability for many addictive behaviors that is implicated by the GARS test. Using GWAS, 265,218 patients and 784,643 controls, the researchers measured the amount of genetic overlap across disorders. 1,191,588 individuals were analyzed for their associations with brain disorders and 17 physical or cognitive measures. Overall, all GWAS consortia identifying sufficient sample sizes and studying common brain disorders were included in the dataset. Psychological disorders and neurological disorders (such as Parkinson’s and Alzheimer’s) appear to share a large number of genetic variants. In contrast to current diagnostic categories, the results suggest that psychiatric disorders share important molecular similarities. In this regard, GARS may have predictive value for addictive behaviors including RDS, not as a diagnostic, but as a test to detect high addiction risk for SUD. The environment, or epigenetics, impacts DNA polymorphisms, in and of itself. According to the mathematical equation P=G=E, the resultant phenotype is the known relationship between these two elements. Psychiatrists and other clinical professionals can use this novel tool to identify people at risk for substance use disorder and a wide variety of RDS behaviors as well.

#### Promoting PAM to combat the global opioid crisis [33]

It is universally acknowledged that dopamine plays a significant role in reward dependence, however, there is controversy regarding how to modulate its role clinically to treat and prevent relapse. Also, most agree that it is important to provide early genetic identification, potentially through a novel technology called GARS. The existing FDA-approved medications promote blocking dopamine; however, a more prudent paradigm shift should be biphasic-short-term blockade and long-term upregulation, enhancing functional connectivity of brain reward networks. Rather than any specific drug or non-drug addictive behavior, RDS is the true phenotype. Therefore, all addictive behaviors have RDS as their true phenotype. To conclude, we propose seriously reconsidering treating RDS by simply supplying powerful narcotic agents (e.g., buprenorphine) in order to combat the current out of control opioid/alcohol crisis worldwide. Addicts will remain addicted to this type of treatment. We call “PAM^™^” a more reasonable solution that involves genetic testing, urine drug screens using CARD and dopamine homeostasis.

#### FOXN3 and GDNF polymorphisms as common genetic factors of substance use and addictive behaviors [34]

A number of addictive behaviors seem to share common underpinnings based on studies in epidemiology and phenomenology. An investigation of genetic overlaps between substance use, addictive behavior, and other compulsive behaviors was conducted as part of the psychological and genetic factors of addictions study (n = 3003). In association analyses, 32 SNPs were examined, along with potentially addictive substances (alcohol, tobacco, cannabis, and other drugs), and potentially addictive and compulsive behaviors (internet usage, gaming, social networking, gambling, exercise, hair pulling, and eating). A nominal significance analysis revealed 29 associations, of which nine survived correction for FDRbl. The FOXN3 rs759364 gene showed four associations with potentially addictive behaviors: alcohol consumption, internet addiction, gaming disorder, and exercise addiction. The “lifetime other drugs” variable was significantly associated with GDNF rs1549250, rs2973033, CNR1 rs806380, and DRD2/ANKK1 rs1800497 variants. There is evidence that genetic factors may contribute similarly to the use of specific substances and to the behavior of addiction. Multiple addictive behaviors may be influenced by FOXN3 rs759364 and GDNF rs1549250 and rs2973033. There is a need for further research because of limitations (e.g., convenience sampling, a lack of structured substance use scales). These relationships should also be examined for functional correlates and mechanisms.

#### Neurophysiological measures and alcohol use disorder (AUD): Hypothesizing links between clinical severity index and molecular neurobiological patterns [35]

Cloninger proposed in 1987 a clinical description of different personality traits that were genetically defined and independent of one another. Furthermore, he devised a test for examining these traits/states, the TCI. There seems to be a direct correlation between high levels of craving and a higher risk of relapse in people with AUD, which has gained increasing significance in recent years. Thus, our study aim is to investigate the possible correlations between TCI-linked molecular neurobiological patterns, craving and alcohol addiction severity measures in a sample of Italian alcoholics. A total of 191 alcoholics were recruited at Sapienza University of Rome in a Day Hospital setting as part of the Alcohol Addiction Program Latium Region Referral Center. The psycho-diagnostic protocol included TCI, VAS-C, ASI, and SADQ after 7 days of detoxification treatment. Informed consent was obtained from all patients by the Institutional Review Board. Our results indicate a significant positive correlation between HA-scale and VAS scores: increasing HA-scale is associated with higher craving perception, both in terms of intensity and frequency (r = 0.310; p = 0.001). SADQ also revealed a significant relationship between perception of dependency severity and both HA (r = 0.24; p = 0.05) and NS (r = 0.24; p = 0.05) scales. With regards to character scales, persistence (r = −0.195; p = 0.008) and self-directedness (r = −0.294; p = 0.001) were negatively correlated with ASI associated with alcohol problems. A negative correlation was also observed between self-directedness and ASI related to family and social problems (r = −0.349; p = 0.001), employment and support (r = −0.220; p = 0.003), and psychiatric problems (r = −0.358; p = 0.001). A negative correlation was found between cooper-ativeness and legal problems (r = −0.173; p = 0.019). A positive correlation was found between self-transcendence and medical problems (r = 0.276; p = 0.001). Our data can suggest that our cohort of patients may be at a particular stage of their addiction history based on recent neurobiological theories such as the “RDS” (including GARS testing) and the Koob model. Therefore, if our hypothesis is confirmed, the TCI-based assessment of alcoholics will allow treatment optimization. As a result of understanding these newer concepts, clinicians may be able to translate this information to their patients and potentially improve clinical outcomes, since it suggests a functional hypothesis of neurotransmitter circuits that helps frame the patient’s addiction history.

#### The molecular neurobiology of twelve steps program and fellowship: Connecting the dots for recovery [36]

A recent statement by the American Society of Addiction Medicine (ASAM) suggests that alcoholism and drug abuse are not diseases at all, but rather consequences of brain disorders. There are some who believe addicts can quit on their own and moderate their alcohol and drug consumption on their own. Many addicts achieve total abstinence after they enter a treatment program or the 12-step program and fellowship. It may, however, be possible to find alternatives that work for certain groups of people when controlled drinking fails. We examine the molecular neurobiological basis of each step of the 12-step program in this expert opinion in order to identify personal differences in recovery. Despite addiction risk gene polymorphisms, the molecular neurobiological basis of the 12 steps can impact RDS. A 2013 Springer Neuroscience Brief explored this topic in part by Blum and others. This expert opinion outlines briefly the molecular neurobiological and genetic links, particularly as they relate to the role of epigenetic changes that are possible in alcoholics anonymous members. In spite of carrying hypodopaminergic polymorphisms (measured by GARS), such as the DRD2 A1 allele, “12 steps programs and fellowships” may induce neuroplasticity and continued DRD2 proliferation. ASAM practitioners recognize the importance of adopting 12-step doctrines for patients who do not have access to the “psychosocial-spiritual trio.” Would it be better to combine dopamine agonist modalities as potential histone-deacetylase activators with the 12 steps? At least science is meeting recovery at a time when joy in recovery can be further redeemed, even if many unanswered questions remain.

#### Lyme and dopaminergic function: Hypothesizing reduced reward deficiency symptomatology by regulating dopamine transmission [37]

Ixodes scapularis is the main vector of Lyme disease in the US. A growing body of evidence indicates that those infected may also suffer from anxiety or depression. Researchers have identified two putative cytosolic sulfotransferases that recognize phenolic monoamines as substrates in these ticks. According to one hypothesis, protracted Lyme disease sequelae may be caused by impaired dopaminergic function in the reward circuits of the brain. In the subsequent recombinant proteins, dopamine and octopamine were shown to be targets of sulfotransferase activity. In itself, this can reduce dopamine production, leading to depression and anxiety associated with RDS. It was shown that Ixosc Sult 1 and Sult 2 could inactivate the salivation signal by sulfonating either dopamine or octopamine in the salivary glands of Ixodid ticks. Anxiety and depression are clinically observed reactions to this infraction. Parkinson’s disease and Lyme disease share many symptoms. As a treatment for Lyme disease, (GARS targeted) pro-dopamine regulation provides the basis for understanding its mechanistic and neurobiological effects on the central nervous system.

#### Withdrawal from Bup/Nal and maintenance with a natural dopaminergic agonist: A cautionary note [38]

Numerous studies demonstrate that methadone and buprenorphine are effective for stabilizing and maintaining opioid dependence, but opioid withdrawal symptoms occur upon tapering and cessation. A 35-year-old Caucasian female (Krissie) who suffered chronic pain from reflex sympathetic dystrophy and fibromyalgia after carpal tunnel surgery is presented here as a case study. After 5 years, the daily dosage increased to over 80 mg of methadone and 300 μg/h. For breakthrough pain, use fentanyl transdermal patches in combination with 12 – 14 1600 μg Actig lollipops and oral 100 mg morphine and 30 mg oxycodone 1 – 2 tabs every 4 – 6 hours. An average of $50,000 was spent each month on prescription drugs, including benzodiazepines (BZDs), hypnotics, and stimulants. A natural dopaminergic agonist, KB220Z, was used during the patient’s inpatient detoxification with Suboxone^®^ in 2008. In follow-up while taking KB220Z daily, we carefully documented her withdrawal symptoms when she abruptly stopped taking Bup/Nal. A reward gene panel was also genotyped on the patient, including (9 genes 18 alleles): DRD2,3,4; MOA-A; COMT; DAT1; 5HTTLLR; OPRM1; and GABRA3. KB220Z is being administered to the patient 432 days after Suboxone^®^ withdrawal, and he has had a urine test confirming that he is free of opioids. According to genotyping results, there is a moderate genetic risk for addiction, indicating a hypodopaminergic trait. Based on these preliminary case data, KB220Z might provide a cost-effective alternative to Suboxone^®^ as an adjunctive therapy. To test the hypothesis that KB220Z may function as an adjunct to natural opioid substitution maintenance, we encourage double-blind randomized controlled trials.

#### Hypothesizing high negative emotionality as a function of GARS testing in AUD [39]

In fact, negative emotionality plays a major role in motivating people to consume alcohol and, ultimately, in relapsing. Since genetic factors are linked to both of these unwanted events, using the GARS test may provide insight into specific genetic antecedents and even epigenetic influences.

#### Polygenic and multi locus heritability of alcoholism: Novel therapeutic targets to overcome psychological deficits [40]

For the GARS^®^ test, ten genes and 11 polymorphisms connected to the promotion of genetically induced hypodopaminergia met the final selection criteria. GARS^®^ test results are affected by hypodopaminergia, a complicated but important condition. A key component of the development of GARS^®^ was identifying studies reporting low dopamine function in association with specific SNPs in reward genes. While there are many possible addiction-related genes as pointed out by Li *et al*. dopaminergic neurotransmitter pathways do not exist in isolation but rather embedded within a complex network of interrelated mesolimbic/pre-frontal Serotonergic-, Cannabinoidergic-, Endorphinergic-, GABAergic-, and Glutaminergic (cholinergic) systems each of which exhibits a unique function within the context of addictive behaviors. For the GARS^®^ genetic panel, some of these interactions and polymorphisms have been correlated with dopamine- and reward regulation.

#### “Dopamine homeostasis” requires balanced polypharmacy: Issue with destructive, powerful dopamine agents to combat America’s drug epidemic [41]

Researchers have observed prolonged neuroplasticity (brain cell repair) in rodents that express the well-researched pro-dopamine regulator KB220 and variants. KB220Z also increases overall brain connectivity volume, increases neuronal dopamine firing, and eliminates lucid dreams in humans over a prolonged period in addition to increasing functional connectivity. Numerous clinical studies have validated the effectiveness of this patented nutrigenomic technology in rebalancing brain chemistry and optimizing dopamine function. Additionally, knockoff marketers with lookalike products could deceive and endanger unsuspecting consumers with false promises. The credibility and reputation of validated, authentic, and ethical products are threatened by products that contain ingredients with potential dangers (for example, combinations of potent D2 agonists including L-DOPA and L-Theanine). We encourage clinicians and neuroscientists to continue to embrace the concept of “dopamine homeostasis” and search for safe, effective, validated, and authentic means to achieve a lifetime of recovery, instead of reverting to anti-dopaminergic agents doomed to fail in the war against the devastating drug epidemic or promoting powerful D2 agonists that compromise needed balance (GARS induced KB220 customization).

#### Pro-dopamine regulator - (KB220) to balance brain reward circuitry in RDS [42]

Currently, we are facing a devastating epidemic of opiates and opioids everywhere in the world. CDC statistics indicate that 127 people die every day from narcotic overdose in America, young and old. As well as opiate/opioids, alcohol and nicotine, MATs have been approved by the FDA. In most MATS, either dopaminergic blockade or opioid substitution therapy are the mechanism of action. While these options are effective for treating addiction symptoms short-term, they fail to address the underlying causes or lead to long-term recovery. The search for better treatment options needs to continue. The purpose of this mini review is to describe the development of KB220, a GDOC. Increasing evidence suggests that brain reward circuitry controls drug addiction, along with “anti-reward systems” since both glutaminergic and dopaminergic transmission can affect anti-reward systems. As a result of KB220 therapy, the brain reward system may eventually be balanced, and dopamine homeostasis may be induced. The addiction science literature incorporates many of these concepts that have been reported elsewhere. This concise review may encourage readers to reconsider these facts and stimulate further research focused on how “dopamine homeostasis” may affect recovery and relapse prevention.

#### Our evolved unique pleasure circuit makes humans different from apes: Reconsideration of data derived from animal studies [43]

It has been proven that certain brain regions are linked to pleasure when we engage in sex, eat tasty food, watch a movie, perform well at school and in athletics, consume drugs, and give to the community, country, and world. According to research, our immune system may be most positively affected by the latter type of satisfaction, supporting the community. The pathways underlying these effects, however, remain unclear. A mesocorticolimbic circuitry well developed by Berridge and Kringelbach is believed to mediate pleasure and serve adaptive functions. As a result of a breakdown in that hedonic system, affective disorders can result in anhedonia (an absence of pleasure) or dysphoria (negative feelings). Neuroimaging studies on humans show that quite diverse pleasures activate similar neural pathways, indicating that all rewarding stimuli and behaviors share a common neural pathway. There has been confusion over the involvement of dopamine in pleasure/reward over the years, for example separating motivation from pure pleasure (i.e., liking versus wanting). Based on self-reports in humans, animal studies cannot provide true clinical information. A new piece of evidence confirming our concerns surfaced on November 23^rd^, 2017. A large research team reported in the journal science that humans developed a brain system that plays a role in everything from addiction to autism. Moreover, Sousa et al.’s findings also suggest that rodents and even non-human primates should not be over-used for studies. The data do not support extrapolations regarding pleasure, dopamine, and reinforcement. There is now a greater emphasis on human experience and study. There may be a higher probability of fiction than fact when extrapolating from non-humans to humans. Despite the boldness of this statement, it should not be construed to suggest that animal data is not important. Animal models for diseases are extremely valuable in many ways, so we should encourage their development. Nevertheless, we must be careful not to jump to conclusions based on results that are not supported by follow-up human experiments. We are further proposing that in order to overcome a never-ending battle related to the current drug epidemic, the scientific community should realize that disturbing dopamine homeostasis by taking drugs or having a system compromised by genes or other epigenetic experiences should be treated with alternative therapeutic modalities, as described in this article as a realistic goal. Application of GARS^™^ testing and pro-dopamine regulation (KB220) should be considered along with other promising technologies including cognitive behavioral therapy, mind fullness, brain spotting and trauma therapy. Basic scientists have worked very hard to dis-entangle pleasure from incentive salience and learning signals in brain reward circuitry, but this work may be limited to animal models and rodents. A different consideration regarding the human reward systems is required.

#### GARS: Molecular neurogenetic evidence for predisposition to RDS [44]

Neurogenetic studies of brain reward systems, especially genes associated with dopaminergic function, have been published extensively. It was in 1996 that we coined the term “Reward Deficiency Syndrome”, to describe behaviors associated with hypodopaminergic dysfunction. Numerous subsequent studies have supported RDS as a useful concept for understanding SUD, addictions, and other obsessive, compulsive, and impulsive behavior. By evaluating the resultant GARS^™^ data only, we were able to describe lifetime RDS behaviors in a recovering addict (17 years sober). To reduce or eliminate pathological substances and behavioral seeking activity, genetic testing at an early age may be an effective preventive strategy. By utilizing GWAS, we demonstrate convergence to reward candidate genes for a select number of genes and their polymorphisms associated with RDS. Based on the evidence presented, targeted therapies can enhance recovery and prevent relapse by providing relevant genetic information. RDS is primarily driven by a hypodopaminergic trait (genes) as well as epigenetic states (methylation and deacetylation of chromatin structure). In addiction medicine, we have now reached a new era based on neuroscience that recognizes RDS as a pathological condition of the brain reward circuitry that requires effective evidence-based therapy, early genetic diagnosis, and further intensive research.

#### Dopamine genetics and function in food and substance abuse [45]

We have entered the genomic era with confidence in the future of medicine, including psychiatry, and have begun to understand how DNA and polymorphic associations affect brain reward circuitry. A major benefit of this strategy may be to treat millions of people suffering from “RDS”, a genetic disorder of the brain’s reward circuitry. The article discusses the relationship between drugs and food addiction, including dopamine genetics and function, and the interaction between sodium food and dopamine transporters. Our concept related to genetic antecedents of multiple addictions (RDS) will be briefly discussed. Stratification of genetic risk to RDS can also be achieved by evaluating a panel of established reward genes and polymorphisms. GARS is a tool for diagnosing a genetic predisposition for RDS and is called the GARS. By identifying at-risk individuals at an early age, this test would benefit the medical community, as pointed out by others. In depth research on both human and animal addiction models is encouraged. The Salted Food Addiction Hypothesis as well as other forward-thinking hypotheses should be explored further in order to understand the neurogenetic correlates of food and drug addiction.

#### Molecular neuro-biological and systemic health benefits of achieving dopamine homeostasis in the face of a catastrophic pandemic (COVID-19): A mechanistic exploration [46]

In the face of the global pandemic of COVID-19, approaching 1.75 million infected worldwide (4/12/2020) and associated mortality (more than 108,000 as of 4/12/2020), as well as other catastrophic events including the opioid crisis, it seems prudent (http://www.coronavirus.gov). RDS dysfunctional conditions and their effects on behavioral physiology, function of reward genes, and the constellation of symptomatic behaviors. This manuscript discusses the systemic benefits of restoring and achieving dopamine homeostasis to reverse and normalize thoughts and behaviors. It will be discussed how nutrigenomic interventions can help restore normal brain function and how they can benefit these systems. Instead of pharmaceutical interventions, we demonstrate that nutrigenomic dopamine agonists can modulate dopamine homeostasis. Free radicals, chronic diseases and disorders, and anaerobic events have all been extensively discussed. Furthermore, dopamine is discussed extensively in connection with sleep, rapid eye movement, and waking. In addition, a special focus is placed on ocular health and the influence of taste sensations on the brain. Additionally, the detailed mechanistic aspects of dopamine, immune competence, and autoimmune disorders are discussed. A research-validated nutrigenomic intervention and a patented gene test (GARS) are presented to integrate dopamine homeostasis. The ability to achieve dopamine homeostasis may prove to be a technological paradigm shift in our understanding of the health benefits of achieving it.

#### Should we embrace the incorporation of genetically guided “dopamine homeostasis” in the treatment of RDS as a front-line therapeutic modality? [47]

Statistics related to drug overdoses in the US were released by the US CDC in 2019. Almost 72,000 deaths in the US were due to opioid overdoses, according to the study. There is an alarming increase in this rate every year. The COVID-19 pandemic, despite the new vaccines, will have an even greater impact on increased drug use in 2021, making it a particularly scary year. In 2020, overdose deaths nationwide increased to 13% and in some states to 30% because of opioid overdoses. The common neuromodulating aspects of neurotransmission, and its disruption via chronic exposure to drugs and behavioral addictions, require more intensive research focusing on developing novel strategies to combat these unwanted genetic and epigenetic offenses, as achieved by our group with heroin addiction. There is a plausible acceptance of the well-established evidence for hypodopaminergia, a blunted reward processing system, reduced functional connectivity at rest, genetic antecedents, anti-reward symptoms, poor compliance with MAT, and generalized RDS. A future approach to dopamine homeostasis should be pursued through intensive future research based on this evidence. Many beneficial modalities may be used to achieve this paradigm shift, including exercise, pro-dopamine regulation, nutrigenomics, cognitive behavioral therapy, hedonic hot spots in the brain, rTMRS, deep brain stimulation, diet, genetic editing, genetic guided therapeutics (GARS), epigenetic repair, among others. A “hypodopaminergic ditch” is what millions of people may be able to escape through nutrigenomics.

#### In search of RDS-free controls: The “holy grail” in genetic addiction risk testing [48]

Based on hundreds of published studies about the role of dopamine in addictive behaviors, including drug dependence and compulsive/impulsive behavior disorders, the search for an accurate, gene-based test to identify heritable risk factors for RDS was conducted. A polymorphic allelic propensity for hypodopaminergia was first associated with RDS in 1995 by Blum’s group. To illustrate how the GARS test is designed to select risk alleles of reward genes. As a result, case-control behavioral association studies are hampered by inconsistent results. Researchers may have failed to adequately screen controls for drug and alcohol use disorders, obesity, pathological gambling, and internet gaming addiction because they failed to screen for drug and AUDs. For selection of alleles to be measured by the GARS test, a review of literature related to the function of reward genes associated with hypodopaminergia relevant case-control association studies was conducted. An example of a possible solution is comparing the prevalence of DRD2 A1 in unscreened controls (33.3%) with “super-controls” (highly screened RDS controls (3.3%) in the proband and family). RDS is polygenetic and very complex, as opposed to one gene-one disease. Additionally, any behaviors associated with RDS must be excluded from the control group in order to obtain the most accurate statistical analysis.

#### Epigenetic repair of terrifying lucid dreams by enhanced brain reward functional connectivity and induction of dopaminergic homeostatic signaling [49]

When one has a lucid dream, they are aware of the dream, experience it as if they are awake, and have control over it. Based on the dream’s nature and pleasantness, the dreamer can start, stop, and restart dreaming. RDS patients may experience a pleasant, unpleasant, or frightening dream content depending on the severity of their disorders. According to a study of patients with RDS identified at a psychiatric center, KB200Z, a dopamine agonist, was effective in combating terrifying, lucid dreams. As a result of these reports, eight clinical cases with substance abuse, childhood abuse, and post-traumatic stress disorder were examined. In 87.5% of cases, KB200Z was found to eliminate unpleasant or terrifying lucid dreams. Further published cases have demonstrated that PTSD and ADHD patients can eliminate terrifying dreams over time. Neurogenetic and epigenetic changes in neuroplasticity, identified in the pathogenesis of PTSD and ADHD, can be mitigated by inducing dopamine homeostasis. This article explores how a neuro-nutrient can modulate dopaminergic signaling to relieve terrifying lucid dreams. In recent years, KB220 neuro-nutrients have been used in precision formulations guided by GARS test results to repair inheritable deficiencies. Positive cognition is aimed to be stimulated by improved dopamine transmodulation signaling, which leads to attenuation of epigenetic insults resulting from trauma as a result.

#### Cannabis-induced hypodopaminergic anhedonia and cognitive decline in humans: Embracing putative induction of dopamine homeostasis [50]

As evidenced by the rise of cannabis use disorder, which is estimated to be 8 percent, the regular use of cannabis among young adults has increased substantially over recent years. There are 3% of Americans who are unemployed. Young adults with cannabis exposure are more likely to suffer from hypodopaminergic anhedonia (depression), cognitive decline, poor memory, inattention, impaired learning performance, reduced emotionality associated with dopamine brain responses, and more likely to become addicted. The addiction medicine community is increasingly concerned because of the high concentration of delta-9-tetrahydrocannabinol (THC) currently found in oral and vaping cannabis products, and the cognitive effects of cannabis may become more pronounced in young adults who use these products. According to preliminary research, “dopamine homeostasis” can be induced with the proposed compound, restoring dopamine function and normalizing behavior in chronic cannabis users with hypodopaminergic anhedonia (depression) and cognitive decline due to cannabis use. A decriminalization policy can be developed using the findings of this psychological, neurobiological, anatomical, genetic, and epigenetic research.

#### The effects of residential dual diagnosis treatment on alcohol abuse [51]

The study involved 804 residents with co-occurring alcohol and mental health disorders in dual diagnosis programs. One, six, and 12-month follow-up assessments were conducted on the ASI. Repeated measures analysis showed the intoxication rate per month stabilized between months six and 12 with 68% still in remission and an 88% mean reduction from baseline (F = 519, p < 0.005). A comparison between patients with and without weekly relapse produced significant differences in hospitalization (odds ratio 11.3:1; 95% C.I., 5.5 to 23.2). Eight ANCOVAs used mean intoxication days per month after discharge as the outcome variable, pre-admission intoxication days per month as a covariate, and eight variables associated with relapse (e.g., depression) as factors [at admission future GARS testing will help predict clinical outcomes]. Patients with these factors at admission did not have significantly higher intoxication rates after discharge than patients without them. This suggests that these DD programs successfully integrated treatment of both disorders and explained their effectiveness. Co-occurring DSM IV mood disorders such as anxiety and depression as well as drug abuse involving opioids or cocaine fell between 66 and 95% at months one, six, and twelve.

#### Molecular neurological correlates of endorphinergic/dopaminergic mechanisms in reward circuitry linked to endorphinergic deficiency syndrome [52]

The current literature strongly supports the concept that brain neurotransmitters, and second messengers involved in the net release of dopamine in the mesolimbic region, particularly the NAc, are directly related to motivation, anti-stress, incentive salience (wanting), and well-being. The role of dopamine in terms of alcohol withdrawal symptomology, cocaine craving behavior, dopamine-condensation products, and more recently, genetic factors influencing drug seeker behavior and pro-dopamine regulation, provides compelling evidence of the relevant molecular neurological correlates of dopaminergic/endorphinergic mechanisms in reward circuitry due to genetic polymorphisms and epigenetic insults. OUD should be treated with lifelong opioid substitution therapy in the face of the American opioid epidemic. However, the authors suggest a paradigm shift involving novel modalities like targeting the endorphinergic system linked to dopamine release at the NAc, in terms of the induction of required “dopamine homeostasis.” Utilizing the known genetic-environmental interaction theorem P = G + E, the authors provide a clear rationale for the adoption of GARS test coupled with endorphinergic/dopamine regulation to address dysfunction across the brain reward circuitry. The goal of altering resting-state, functional connectivity may require a gentle “neurotransmitter fix” vis enkephalinase inhibition to overcome or combat - self-induction of acute dopamine release via psychoactive substance misuse resulting in chronic dopamine down-regulation. As subsets of reward deficiency, we are poised to provide novel, genetically guided therapy for endorphinergic, opioidergic, and dopaminergic deficiencies and related syndromes, utilizing “PAM”.

#### Endorphinergic enhancement attenuation of PTSD via activation of neuro-immunological function in the face of a viral pandemic [53]

SUD and PTSD predisposition can be associated with polymorphic gene variants, particularly genetic determinants of low dopamine function (hypodopaminergia). National Institutes of Health (NIH) addiction research and molecular genetic technologies have revealed the importance of reward circuitry in addiction and PTSD symptoms. Israeli researchers found that mice lacking adaptive immunity were more likely to develop PTSD when compared with mice with a normal immune system. Raise endorphinergic function and significantly increase immune response is well established. D-phenylalanine, an enkephalinase inhibitor, increases brain endorphins in animal models and reduces stress in humans, according to Blum’s research. PTSD is treated by restoring endorphin function with enkephalinase inhibition with D-phenylalanine. In terms of genetic risk for stress vulnerability versus resilience, GARS can be used to determine relevant phenotypes. PTSD can be managed with customized neuro-nutrient supplements following return from deployment by using GARS as part of pre-testing military enlistees for adaptive immunity. Dopamine homeostasis may be restored by pro-dopamine regulation based on GARS values, particularly when focusing on improving immunological function. Stress disorders may be treated differently if the immune system is recognized as a “sixth sense” and adaptive immunity is assisted with PBM, in conjunction with other supportive interventions and therapies.

#### A review of DNA risk alleles to determine epigenetic repair of mRNA expression to prove therapeutic effectiveness in RDS: Embracing “PBM” [54]

This is a review of research on “PBM” of SUD. America is experiencing a high prevalence of SUD, primarily involving legal and illegal opioid use. A 3000% increase in treatment for substance abuse occurred between 2000 and 2016. Unfortunately, present day treatment of opioid abuse involves providing replacement therapy with powerful opioids to, at best, induce harm reduction, not prophylaxis. These interventions do not enhance gene expression and restore the balance of the brain reward system’s neurotransmitters. We are proposing a generalized approach called “PBM.” This approach includes: (1) using the GARS (a 10 candidate polymorphic gene panel shown to predict ASI-alcohol and drug severity) to assess early pre-disposition to substance use disorder; (2) using a validated RDS questionnaire; (3) utilization of the CARD^™^ to assess treatment compliance and abstinence from illicit drugs during treatment, and, importantly; (4) utilization of a “pro-dopamine regulator (KB220)” (via IV or oral (KB220Z) delivery systems) to optimize gene expression, restore the balance of the BRC’s neurotransmitter systems and prevent relapse by induction of dopamine homeostasis; and (5) utilization of targeted DNA polymorphic reward genes to direct mRNA genetic expression profiling during the treatment process. Incorporation of these events can be applied to not only the under-considered African-American RDS community, but all victims of RDS, as a demonstration of a paradigm shift that uniquely provides a novel putative “standard of care” based on DNA guided precision nutrition therapy to induce “dopamine homeostasis” and rebalance neurotransmitters in the BRC. We are also developing an RDS Diagnostic Criteria to assist in potential tertiary treatment.

#### Hypodopaminergia and “PBM”: It is a generational family affair [55]

The proband was a female with a history of drug abuse and alcoholism. A customized pro-dopamine regulator was matched to polymorphic reward genes with hypodopaminergic risk. Under the influence, she was involved in a car accident and voluntarily sought treatment. Based on the identified polymorphisms in the GARS, she was genotyped and started a neuro-nutrient with a KB220 base. It was prescribed to her in April 2018, and she is still taking it. After overcoming SUD, she improved her socialization, family, economic status, well-being, and attenuated major depression. A recent screening and her first two months of treatment both came back negative. The GARS variants were recommended to her parents approximately two months after she started taking them. The proband’s father (a binge drinker) and mother (without SUD) improved their behavioral issues. A high risk for SUD was also found in the proband’s biological children who were also GARS tested. In this three-generation case series, genetic information combined with a DNA-guided “pro-dopamine regulator” is shown to enhance life quality and aid in recovery.

#### Exploration of epigenetic state hyperdopaminergia (Surfeit) and genetic trait hypodopaminergia (Deficit) during adolescent brain development [56]

Especially in adolescents with unmyelinated prefrontal cortex, drug and non-drug addictions pose important and complex risks. Epigenetics plays a key role in the brain development of adolescents, compared to adults, in numerous animal and human studies. There is evidence of underlying hyperdopaminergia causing reward site neurons to release high levels of presynaptic dopamine, which can set our youth up for risky behaviors. Furthermore, altered reward gene expression caused epigenetically by social defeat, like bullying, can persist into adulthood. Conversely, epigenetic events may also cause adolescent hypodopaminergia. As a result of this complexity, neuroscience cannot definitively conclude that all adolescents are hyperdopaminergic. Here, the primary issue is whether this population suffers from mixed hypo- or hyper-dopaminergia. 24 Caucasians with RDS, ages 12 – 19, were tested for GARS^®^. This cohort of adolescents with RDS parents displayed a high risk for any addictive behavior (hypodopaminergia), especially drug-seeking (95%) and alcohol-seeking (64%). Although more work is needed, adolescents in our study exhibit a hypodopaminergic trait, a result of a family history of RDS. It is certain that we will analyze GARS in non-RDS Caucasians between the ages of 12 – 19 in future studies. A well-researched, precise, pro-dopamine nutraceutical regulation is suggested first, followed by the identification of risk alleles using the GARS test. As a result of the American opioid/psychostimulant epidemic, this “two-hit” approach might prevent tragic deaths among adolescents.

#### Coupling GARS and pro- dopamine regulation (KB220) to combat SUD [57]

The systematic medical approach to reward transformation (SMART^™^) is a generalized approach based on the RDS. A validated RDS questionnaire is used to diagnose early predisposition (even in children) using the GARS; urine drug testing during actual treatment based on a comprehensive analysis of reported drugs to determine compliance with prescribed medications as well as non-abstinence illicit drugs; and adjunctive treatment with glutaminergic-dopaminergic optimization nutraceuticals (KB220) in order to prevent relapse by enhancing dopamine homeostasis and preventing relapses. In recent placebo-controlled resting state functional connectivity magnetic resonance imaging (rsfMRI) experiments in the rat, KB220Z was shown to enhance resting connectivity between reward and cognitive brain regions. Additionally, KB220Z modulates anterior cingulate cortex theta power in humans. A number of double-blind controlled trials have shown that KB220Z improves craving attenuation and relapse prevention in obese patients with the D2 receptor A1 allele compared to obese patients with the usual complement of D2 receptors. As a result of epigenetically changing the neuro-mechanisms which control dopamine homeostasis, KB220Z may offer clinical benefit in the present article application. New strategies are needed to treat RDS based on this research and current literature. Relapse prevention and effective recovery from psychoactive substance abuse have been limited by traditional therapeutic agents that have failed to address the reduced connectivity patterns in many addictions. This concept would enable genetic testing and precise treatment of genetic and epigenetic deficits with formulations of KB220. Recovery and good health would be the goal of this SMART^™^, holistic program design, based on this extensive research in a diverse but stable population. In a diverse but stable population like African-Americans as well as other minority groups showing high genetic risk for all RDS behaviors.

#### Hypothesizing nutrigenomic-based precision anti-obesity treatment and prophylaxis: Should we be targeting sarcopenia induced brain dysfunction? [58]

A total obesity rate of 30% is estimated for 12 states by the US CDC and is estimated to be 20% nationwide. Despite preventative measures implemented throughout the world, the obesity epidemic persists. There is a promise that pharmaceutical treatments will reduce total fat mass. The side effects of medications can, however, be serious and may even prove fatal. Based on a PubMed search of the key term’s “obesity” and “sarcopenia,” this review presents evidence corroboration of the existence of RDS in obesity and the involvement of catecholaminergic pathways in substance seeking behavior, especially carbohydrate cravings. It is considered a prevention method to identify the genetic basis for aberrant generalized craving behaviors and to test children for those risks in the future. Using precursor amino acids therapy and modulating the levels of enkephalinase, MOA, and COMT in key brain regions has been demonstrated to be effective. The effects of these treatments are manifested in higher levels of dopamine/norepinephrine, GABA, serotonin, and enkephalins in the brain. In addition, we present evidence supporting the impact of chromium salts on dopamine neuronal synthesis regulation on insulin sensitivity. It is our belief that our unique combination of natural ingredients will promote a healthy metabolism and well-being. In addition to reducing angiogenesis, sarcopenia may also reduce cerebral blood flow. This obesity-related loss of muscle density appears to be overcome by exercise. A proposed nutrigenomic formula based on genetic obesity risk testing can induce significant healthy fat loss and prevent relapses by inhibiting carbohydrate bingeing and promoting generalized anti-craving of carbohydrates.

#### Death by opioids: Are there non-addictive scientific solutions [59]

The current opioid crisis in America has caused close to 800,000 premature deaths since 2004, so we propose a novel approach in an effort to reduce this number. The governmental institutes and professional societies (NIDA, NIAAA, ASAM, and American Board of Addiction Medicine (ABAM)) are making extraordinary efforts to combat this continuing dilemma, but the current approach is failing, and alternatives should at least be explored. For researchers, clinicians, and counselors treating RDS, these truths present a serious ethical dilemma. Despite DSM-V’s claims, brain reward is not accurately described in the current version. It is not possible for the human brain to perceive addictions such as gambling as distinct endophenotypes like opioids, alcohol, nicotine, cocaine, BZDs, or cannabis. No matter whether cannabinoids, endorphins, or even BZDs are natural ligands, this is true. It is indeed reward dysfunction (hypodopaminergic or hyperdopaminergic) that is the most accurate endophenotype. Considering this, we propose that the current MATs be extended to needy individuals as a short-term “band-aid” to reduce harm avoidance, and a long-term “prophylactic” measure. In addition to MAT, there might be other promising treatments, including repetitive transcranial magnetic stimulation, exercise, and even new medications that modulate GABA-A receptors positively allosterically, as well as the highly researched GARS paired with precision KB220Z. Induced “dopamine homeostasis” will help rebalance and restore healthier brain function by promoting the cross-talk between brain regions (such as the NAc, cingulate gyrus, hippocampus, etc.). By providing scientifically sound natural alternatives to addictive substances, we aim to not only save lives, but also improve the quality of life of recovery communities.

#### RDS: A cytoarchitectural common neurobiological trait of all addictions [60]

Comorbidity exists between alcoholism and other substance use disorders, i.e., a reduction in dopamine signaling within the reward pathway. The term RDS refers to behaviors associated with addiction, obsessive compulsiveness, and impulsivity. The number of people who suffer from opioid use disorder and are dependent on prescription opioids in the US is estimated to be 2 million. Over one trillion dollars are estimated to be spent on the illegal and legally prescribed opioid crisis. Most addictions and RDS disorders are treated with opioid replacement therapy. Despite repeated relapses, opioid replacement treatments are prescribed to patients repeatedly. According to a recent report in JAMA, non-opioid treatments are more effective than chronic opioid medications. Alcohol and other drugs are involved in more than half of all suicides, according to research. Furthermore, nutrigenomic therapies (e.g., KB220Z) optimize gene expression, rebalance neurotransmitters, and restore neurotransmitter function. Dopaminergic function was shown to be enhanced by KB220Z across specific brain regions. A significant reduction in RDS behavior disorders and relapses has been found with KB220/Z in human DUI offenders. The KB220Z semi-customized nutrigenomic supplement restores dopamine homeostasis effectively when combined with the GARS test.

#### Researching mitigation of alcohol binge drinking in polydrug abuse: KCNK13 and RASGRF2 gene(s) risk polymorphisms coupled with GARS guiding precision pro-dopamine regulation [61]

In the US and throughout the world, excessive alcohol intake, e.g., binge drinking, is a significant and mounting public health issue. In order to improve prevention and therapeutic strategies, we need a better understanding of the underlying neurobiology. Our group defined the reward deficiency domains of alcoholism and other substance use disorders based on the reduced dopamine signaling of reward pathways and the restoration of them with specifically designed therapeutic compounds by using a darkness-induced alcohol intake protocol. The genes KCNK13 and RASGRF2 are encoded for potassium two pore domain channel subfamily K member 13 and Ras-specific guanine nucleotide-releasing factor 2, respectively, which are associated with alcohol binge drinking and have important dopamine-related functions. Based on GARS guided precision pro-dopamine regulation, we hypothesize that KCNK13 and RASGRF2 genes’ risk polymorphisms may mitigate binge drinking. The purpose of this review is to provide data on the benefits of this unique approach for both binge-drinking animals and drunk drivers, including reducing alcohol intake and preventing relapses. There is growing evidence to support the use of GARS with or without KCNK13 and RASGRF2 risk polymorphism in the legal arena, since driving under the influence often results in incarceration rather than rehabilitation. As a defense strategy, the argument that “determinism” overrides free will may be plausible. A major problem related to polydrug abuse can be resolved through this type of research.

#### Hypothesizing in the face of the opioid crisis coupling GARS testing with electrotherapeutic nonopioid modalities such as H-Wave could attenuate both pain and hedonic addictive behaviors [62]

Despite the opioid overdose epidemic in the US, nonaddictive/nonpharmacological proven strategies can effectively manage chronic pain without opioids. In American chronic pain management, evidence demonstrating opioids’ long-term effectiveness is lacking, as is the desire to change the drug-embracing culture. The “addictive brain” is biologically induced into some pain clinicians by classical analgesic agents, which promote unwanted tolerance to analgesics. As part of the dopaminergic circuitry, reward genes play an essential role in modulating nociception. As a result, chronic pain syndromes may be affected on several sensory and affective levels. It may be possible to reduce pain and prevent addiction with the GARS test and the H-Wave test at entry into pain clinics. Using the GARS test results, high-risk patients can be identified for both drug and alcohol abuse, and H-Wave can be used to treat pain instead of opioids. Despite the need for randomized control studies, H-Wave can be utilized to alleviate pain and reduce hedonic addictions. With this frontline approach, potent opioid analgesics would be less likely to cause long-term neurobiological deficits and fatalities.

#### The benefits of GARS^™^ testing in SUD [63]

The GARS was developed based on 25 years of extensive research by many scientists around the world. In unpublished research, GARS significantly predicted alcohol and drug dependency severity when compared with the ASI, which has been used in many clinical settings. As 127 young and old people die daily from opiate/opioid overdoses, as a result of early testing for addiction and other RDS subtypes, parents are in need of assistance. Addiction to opiates has caused many families to be in real danger and to lose loved ones. As he travelled around the US, Bill Moyers noted that many children with ADHD and other spectrum disorders like Autism also had related conditions such as substance abuse. Rather than prescribing addictive pharmaceuticals to these children, he urged better ways to identify them and treat them. The GARS gene panel is the only one with established polymorphisms reflecting the BRC, which correlates with the ASI-MV risk severity score. GARS provides clinicians with a non-invasive genetic test that can be further validated and extended to include other hypodopaminergic genes and polymorphisms. Clinical interactions and decision-making can be improved with genomic testing, such as GARS. Knowledge of precise polymorphic associations can help in the attenuation of guilt and denial, corroboration of family gene-o-grams; assistance in risk-severity-based decisions about appropriate therapies, including pain medications and risk for addiction; choice of the appropriate level of care placement (i.e., inpatient, outpatient, intensive outpatient, residential); determination of the length of stay in treatment; determination of genetic severity-based relapse and recovery liability and vulnerability; determination of pharmacogenetic medical monitoring for better clinical outcomes (e.g., the A1 allele of the Consequently, naltrexone’s clinical effectiveness is reduced by a decrease in opioid delta receptor binding by the DRD2 gene; and supporting medical necessity for insurance scrutiny.

#### Understanding the scientific basis of PTSD: PBM overrides stigmatization [64]

A polygenic disorder, PTSD, is triggered by environmental factors. PTSD and SUDs share genetic predispositions to hypodopaminergia (low dopamine function), which is the result of polymorphic genes. Researchers at the NIH have developed new innovative approaches to early diagnosis and treatment of some PTSD symptomatology and addiction thanks to neuroimaging research and molecular genetic applied technologies. These approaches have improved understanding of brain reward circuitry functions. In this review, we present psychosocial and genetic evidence that dopamine regulation may influence resilience or vulnerability to PTSD. The neurotransmitter dopamine is widely recognized as one of the most important in neuroscience. Questions about how to modulate dopamine clinically in order to treat and prevent PTSD and other types of reward deficiency disorders remain. Identification of genetic variations associated with the relevant genotype-phenotype relationships can be characterized using the GARS^®^ and psychosocial tools. Development of an advanced genetic panel is under study and will be based on a new array of genes linked to PTSD. However, for now, the recommendation is that enlistees for military duty be given the opportunity to voluntarily pre-test for risk of PTSD with GARS, before exposure to environmental triggers or upon return from deployment as part of PTSD management. Dopamine homeostasis may be achieved via customization of neuro-nutrient supplementation “PBM^™^” based on GARS test values and other pro-dopamine regulation interventions like exercise, mindfulness, biosensor tracking, and meditation.

#### The benefits of GARS^®^ and pro-dopamine regulation in combating suicide in the American Indian population [65]

The prevalence of alcoholism and other drugs of abuse among Native Americans is well known. In addition, Native Americans also have a high suicide rate compared to other ethnic groups. Suicide rates are also higher in individuals with various psychiatric disorders (e.g., depression) who drink alcohol frequently. There is a four-fold increase in the risk of suicide among males compared to females. Native suicides are more likely to involve alcohol than suicides by local, non-Native people, according to studies comparing Native to other populations in North America. In the US, suicide is the eighth leading cause of death, and the third cause of death for those aged 15 – 24. Despite these disappointing statistics, we propose that, due to a high genetic risk as documented by Barr and Kidd showing that 86% of Native Americans carry the DRD2 A1 allele, compared to only 3% of the highly screened control group. It seems reasonable that early identification, especially in children, be tested with the GARS and concurrently offered the precision pro-dopamine regulator (KB220PAM), which matches their unique brain polymorphisms involving serotonergic, endorphinergic, glutaminergic, gabaergic, and dopaminergic pathways. As a prophylactic measure for reducing substance craving at an early age, PAM may reduce relapses and mortality in adults using the PAM platform.

#### High GARS in chronically prescribed severe chronic opioid probands attending multi-pain clinics: An open clinical pilot trial [66]

Pain affects millions of Americans every day. The number of opioid-related deaths increased from 64,000 in 2017 to 84,000 in 2020, resulting in a decrease in national life expectancy. Chronic opioid use leads to dependence, drug tolerance, neuroadaptation, hyperalgesia, potentially addictive behaviors, or RDS resulting from hypodopaminergia. In a pain clinic study, GARS scores equal to or greater than four and seven alleles significantly predicted drug and alcohol severity, respectively, when compared with the ASI-V. Our study examined the role of eleven alleles in a ten-reward gene panel, reflecting the activity of brain reward circuitry in 121 chronic opioid users, using RT-PCR for SNP genotyping and multiplex PCR/capillary electrophoresis for fragment analysis. There were 55 males and 66 females in the study, with an average age of 54 and 53, respectively. There were Caucasians, African-Americans, Hispanics, and Asians among the patients. A 12-month treatment period for chronic pain was defined as 30 – 600 mg of morphine milligram equivalent for males, and 20 to 180 mg for females. Sixty-six percent of the participants possessed four or more risk alleles, and seventy-three percent possessed seven or more alleles, suggesting a high prevalence of opioid and alcohol dependence. Drug and alcohol addiction is highly prevalent among chronic, legally prescribed opioid users who attend pain clinics. A GARS test may prevent iatrogenic opioid dependence by identifying genetic risk at the time of entry to treatment.

#### Would induction of dopamine homeostasis via coupling GARS^®^ and pro-dopamine regulation benefit benzodiazepine use disorder (BUD)? [67]

There has been an increase in prescriptions for BZDs. In 1999, there was an increase in the use of BZDs alongside opioids, according to national statistics. The brain’s inhibitory neurotransmitter GABA is elevated by BZDs (sometimes called “benzos”). As far as neurochemistry is concerned, BZDs inhibit excitatory neurons by acting at GABA-A receptors, reducing glutaminergic drive at the NAc and reducing dopamine release. It is challenging to treat BUD, partly because BZDs are used to reduce anxiety, which paradoxically induces hypodopaminergia. The paradigm shift we propose is based on this consideration. We propose inducing dopamine homeostasis instead of replacing or substituting chloride channel GABA-A receptors. At least in adults, dopaminergic dysfunction and heightened stress are the root causes of drug and non-drug addictions (i.e., RDS). As part of this proposal, the GARS is coupled with a polymorphic matched genetic customized pro-dopamine regulator called the KB220ZPBM (PBM). It is clinically beneficial to induce dopamine homeostasis during detoxification of alcoholism for at least three reasons: (1) Dopamine regulation during detoxification reduces the need for BZDs; (2) Stress reduction is a major reason for BZDs abuse, so induction of dopamine regulation could be a stress-reducing measure; and (3) It has been shown that BUD and OUD reduce functional connectivity at rest, and as such, potential induction of dopamine regulation enhances resting state functional connectivity. This forward-looking, novel approach will be investigated in future randomized placebo-controlled trials.

#### Coupling neurogenetics (GARS^™^) and a nutrigenomic based dopaminergic agonist to treat RDS: Targeting polymorphic reward genes for carbohydrate addiction algorithms [68]

It was our lab’s earlier research that demonstrated the anti-addiction properties of a nutraceutical containing amino acids precursors and enkephalinase inhibitors, as well as the discovery of the first polymorphic gene associated with severe alcoholism (DRD2) that laid the foundation for “Personalized Medicine.” Our BRC concept has long been used to stratify addiction risk through neurogenetics, even before the later genetic finding. To define a common genetic rubric for both substance-related and non-substance-related addictive behaviors, our laboratory coined the term “Reward Deficiency Syndrome” in 1996. KB220 (Neuroadaptogen-amino-acid therapy (NAAT)) was customized by specific algorithms using polymorphic targets of a number of reward genes (serotonergic, opioidergic, GABAergic, and dopaminergic). The Dutch study identified 1,000 obese subjects and administered KB220Z formulae customized according to DNA polymorphisms individualized, which translated into significant decreases in both body mass index (BMI) and weight in pounds. We have developed a genetic panel of genes known as GARSpDx^™^ based on the results of these experiments. Following the selection of 10 genes with appropriate variants, a statistically significant correlation was found between the ASI-V-alcohol and drug severity scores and GARSpDx. A variant of KB220Z in abstinent heroin addicts increased resting state functional connectivity in a putative network involving the dorsal anterior cingulate, medial frontal gyrus, NAc, posterior cingulate, occipital cortical areas, and cerebellum. KB220Z also significantly activates seed regions of interest, above placebo, including the left NAc, cingulate gyrus, anterior thalamus, hippocampus, pre-limbic, and infralimbic regions. The brain reward circuitry of KB220Z shows significant functional connectivity, increased brain volume recruitment, and enhanced dopaminergic function. By utilizing GARSDx, we propose a RDS Solution that facilitates early identification of risk alleles and stratification of risk groups. As an algorithmic function of carrying these polymorphic DNA-SNPs, KB220Z ingredients can be altered to target these risk alleles with customized nutrigenomic targeting, potentially leading to the first nutrigenomic solution for addiction and pain ever.

#### GARS, a predictor of vulnerability to opioid dependence [3]

It is known as the BRC because it is the interaction between neurotransmitters and genes that control dopamine release. Individuals with genetic or epigenetic variations within the BRC are at risk for addiction and altered pain tolerance. Scientists and clinicians from a variety of fields discuss GARS, the first test to predict vulnerability to pain, addiction, and other compulsive behaviors. Using dopaminergic tone in pain pathways, innovative strategies are proposed for combating opioid abuse, iatrogenic prescription drug abuse, and mortality. Genetic polymorphisms associated with predisposition to pain vulnerability or tolerance may reside in the mesolimbic projection system. In addition to assisting in the treatment of pain, they can identify risk factors for addiction in the future. The pharmacogenomic testing of candidate genes such as type-1 cannabinoid (CB_1_), mu receptors, and PENK may lead to improved clinical outcomes and pharmacogenomic, personalized solutions. A genetic test could provide municipalities with a frontline tool to provide better resource allocation, especially in populations at risk for RDS.

#### Neurobiology and spirituality in addiction recovery [69]

To better cope with the ever-increasing catastrophes that face humanity, is it feasible or desirable to apply genetic engineering to increase human spiritual and religious experience - (gene-spirituality)? The mirror neuron system, the default mode network, and the reward deficiencies (RDS) (hypodopaminergia) are examined as neural connections between spirituality and reward genes. A cornerstone of PMLR is the neuro-spiritual connectome, which may be enhanced by some addiction medicine interventions.

#### Psychoactive drugs like cannabis induce hypodopaminergic anhedonia and neuropsychological dysfunction in humans: Putative induction of dopamine homeostasis via coupling of GARS testing and precision pro-dopamine regulation (KB220) [70]

Many US states now embrace the medical and recreational use of cannabis. Changes in the laws have heightened interest and encouraged research into both cannabinoid products and the potential harms of Cannabis use, addiction, and intoxication. The major active ingredient of *Cannabis sativa* (marijuana), THC and it powerfully stimulates the CB_1_ receptor. When used in the form of plant marijuana, because of the many compounds that exist in the plant form they could inhibit the activity of the CB_1_ receptor thereby reducing many of the effects of THC. While this mechanism seems correct, in our opinion, Vallee et al. incorrectly suggest that blocking CB_1_ receptors could open unforeseen approaches to the treatment of cannabis intoxication and addiction. We caution the scientific community that other CB_1_ receptor blockers, such as, rimonabant (SR141718) have been pulled off the market in Europe. In addition, CB_1_ receptor blockers were rejected by the FDA due to mood changes including suicide ideation. We argue that one issue facing the scientific community, has to do with the increasing legalization of cannabis products in many states across America. We are in favor of some reform in terms of either decriminalization or restrictive legalization, especially in control of legal limits of THC. Like other psychoactive compounds at high doses, it is our hypothesis that chronic use of these drugs including high THC content in its various forms (wax, smoke, or vapor) resulting in brain reward dysfunction induces an imbalance of neurotransmission and subsequent hypodopaminergia and lead to aberrant substance and non-substance (behavioral) addictions. It is further proposed that in order to overcome THC and even other psychoactive drugs of abuse induced anhedonia the coupling of GARS and pro-dopamine regulation is warranted.

#### A novel precision approach to overcome the “addiction pandemic” by incorporating GARS and dopamine homeostasis restoration [71]

To induce dopamine homeostasis for detoxification and treatment of individuals genetically predisposed to RDS, this article describes a novel therapeutic precision intervention combining enkephalinase inhibitors, enkephalin, and dopamine-releasing neuro-nutrients. GARS test results are used to formulate the formulations. It evaluates reward genes and risk alleles based on both neurogenetic and epigenetic evidence. Evidence suggests that people in recovery who are at risk for developing OUD related risks can benefit from the novel genetic risk testing system. Often, opioid use disorders are caused by long-term maintenance agonist treatments such as methadone and buprenorphine, or they may have existed but were unrecognized. Furthermore, the test will assess whether medication-assisted treatment with dopamine augmentation can be beneficial. Through anti-reward allostatic neuroadaptations, RDS methodology has the potential to reduce the burden of addictive disorders on individuals, their families, and society at large.

#### Biotechnical development of GARS and selective evidence for inclusion of polymorphic allelic risk in SUD [72]

Drug and non-drug addictive, obsessive and compulsive behaviors are all included in research on the neurogenetic basis of addiction. The purpose of this paper is to propose a new model for the prevention and treatment of SUD, a subset of RDS behaviors, based on objective biologic evidence. Based on seminal research published in JAMA in 1990, Blum’s group identified the first genetic association with severe alcoholism, which led to the development of the GARS. There have been numerous studies using case-controls that have eliminated SUD despite the fact that no adequate RDS-free controls have been provided to date. It is our contention that this deficiency needs to be addressed in the field, resulting in a reduction in confusion about the role of genetics in addiction if adopted effectively. While not representative of all association studies conducted to date, this sample of case-control studies indicates significant associations between alcohol and drug risk based on the previous literature results presented here. From the current literature, we present 110,241 cases and 122,525 controls. While we may take argument concerning many of these so-called controls (e.g., blood donors), we strongly suggest that it is quite remarkable that a plethora of case-control studies demonstrate selective association with these risk alleles (measured in GARS), most of which indicate hypodopaminergic symptoms. A detailed description of the GARS methodology is presented in the paper. This paper describes the data collection procedures, instrumentation, and analytical approach used to collect GARS and to determine the research objectives. Dopamine homeostasis can be inducted early in the addiction field to combat SUD through genetic risk screening? With the addition of additional scientific evidence, including a future meta-analysis of all available data, a GARS-type screening process will offer a novel opportunity to identify causal pathways and associated mechanisms of genetic factors, psychological characteristics, and addictions.

#### Neuro-genetics of RDS as the root cause of “addiction transfer”: A new phenomenon common after bariatric surgery [73]

Now after many years of successful bariatric (weight-loss) surgeries directed at the obesity epidemic clinicians are reporting that some patients are replacing compulsive overeating with newly acquired compulsive disorders such as alcoholism, gambling, drugs, and other addictions like compulsive shopping and exercise. This review article explores evidence from psychiatric genetic animal and human studies that link compulsive overeating and other compulsive disorders to explain the phenomenon of addiction transfer. Possibly due to neurochemical similarities, overeating and obesity may act as protective factors reducing drug reward and addictive behaviors. In animal models of addiction withdrawal from sugar induces imbalances in the neurotransmitters, acetylcholine and dopamine, similar to opiate withdrawal. Many human neuroimaging studies have supported the concept of linking food craving to drug craving behavior. Previously our laboratory coined the term “Reward Deficiency Syndrome” for common genetic determinants in predicting addictive disorders and reported that the predictive value for future RDS behaviors in subjects carrying the DRD2 Taq A1 allele was 74%. While poly genes play a role in RDS, we have also inferred that disruptions in dopamine function may predispose certain individuals to addictive behaviors and obesity. It is now known that family history of alcoholism is a significant obesity risk factor. Therefore, we hypothesize here that RDS is the root cause of substituting food addiction for other dependencies and potentially explains this recently described Phenomenon (addiction transfer) common after bariatric surgery.

#### Statistical validation of risk alleles in GARS test: Early identification of risk for AUD in 74,566 case-control subjects [74]

We published our study in JAMA in 1990 linking the DRD2 Taq A1 allele to severe alcoholism, and there has been an explosion of genetic candidate association studies, including GWAS. As part of its efforts to develop an accurate test for identifying those at risk for AUD, Blum’s group developed the GARS Test, which consists of ten genes and eleven risk alleles associated with AUD. We applied strict analysis to studies that investigated the association of each polymorphism with AUD or AUD-related conditions published since 1990 in order to statistically validate the selection of these risk alleles measured by GARS. Based on the Hardy-Weinberg equilibrium of each polymorphism in the cases and controls, the Hardy-Weinberg Equilibrium was calculated. Pearson’s *χ*^*2*^ test or Fisher’s exact test was applied to comparisons the gender, genotype, and allele distribution if available. There was a significant finding of OR, 95% CI for OR, and post-risk for 8% estimation of alcoholism prevalence based on the statistical analyses. There was significant OR for DRD2, DRD3, DRD4, DAT1, COMT, OPRM1, and 5HTT at 5%. In spite of the fact that most of the GARS research is derived from our laboratory, we are encouraging more independent research to confirm our findings.

#### Criterion validity of the GARS as a marker of reward deficiency in chemical substances’ addiction: A multi-center study [75]

There is a consistent link between addictive disorders and altered mesolimbic dopaminergic circuits in the brain. This entity’s discussion remains uniformed because it is uncertain if these changes are genetic or acquired. This led us to develop a GARS panel, which includes eleven polymorphisms in ten genes related to dopaminergic reward mechanisms. The DRD1, DRD2, DRD3, DRD4, OPRM1, and COMT genes have six single nucleotide polymorphisms, the 5HTT, DAT1, DRD4, and MAOA transporter genes have four simple sequence repeats, and the GABRA3 gene has a dinucleotide polymorphism. We measured the GARS^™^ criterion against the multimedia ASI-MV in 393 polydrug abusers. There was a significant correlation between GARS and ASI-MV alcohol severity scores. High drug severity also increased GARS, but not linearly. Sequence variation in multiple genes regulating dopaminergic signaling, as well as age, were significant covariates. A reward gene polymorphism that moderately reduced dopamine signaling resulted in a higher ASI-MV score. Drug severity scores are higher when reward-gene polymorphisms moderate dopamine signaling. A preexisting polygenic risk factor may be modulated by pathophysiological and environmental factors related to aging, as suggested by this study that dopamine is implicated in alcoholism and drug abuse. It is important to investigate the corresponding endophenotypes, particularly the role of the hypofunctional dopaminergic system in the RDS.

#### A personalized medicine approach to improve bariatric surgery outcomes utilizing psychosocial and genetic risk assessments [76]

The purpose of this study was to evaluate the potential use of genetic data regarding RDS in predicting negative outcomes in patients undergoing bariatric surgery. A total of 34 patients undergoing bariatric surgery were evaluated for GARS (measures the presence of alleles associated with RDS); as well as their psychosocial traits (questionnaires). Subjects showed an average BMI of 10 kg/m^2^ and an excess weight loss rate of 52 percent. Out of the 11 risk alleles analyzed, the DRD4 risk allele (rs1800955) was significantly correlated with change in weight (r = 0.4726, p < 0.05) and change in BMI (r = 0.4577, p < 0.05) at 6 months post-surgery. A significant negative correlation was seen between the COMT risk allele and EEI scores (r = −0.4983, p < 0.05) and a positive correlation between the GABRB3 risk allele and EEI scores (r = 0.6161, p < 0.01). Scores on the FCQ were also significantly impacted by the GABRB3 risk allele (r = 0.6373, p = 0.001). A significant correlation was found between the PSQI score and the COMT risk allele (r = −0.5482, p = 0.05). An OPRM1 risk allele was significantly correlated with DERS scores (r = 0.5228, p < 0.05). Similarly, DERS scores were significantly correlated with patient BMI before surgery (r = −0.5142, p = 0.01) and six months after surgery (r = −0.6137, p = 0.01). MAOA risk alleles correlated significantly with CESD scores at 6 months (r = −0.4808, p = 0.05). A personalized medicine approach may include genetic testing for obesity and bariatric surgery.

### KB220

#### Acute intravenous Synaptamine Complex Variant KB220^™^ “normalizes” neurological dysregulation in patients during protracted abstinence from alcohol and opiates as observed using qEEG and genetic analysis for reward polymorphisms: Part 1, pilot study with 2 case reports [77]

People who are addicted to food or drugs are more likely to develop dopamine resistance because of the A1 allele of the DRD2 gene. It is becoming apparent that there is potential for utilizing a natural, nonaddictive, safe, putative D2 agonist in the recovery of patients addicted to psychoactive chemicals from RDS. We investigate the potential activating effects of Synaptamine Complex Variant KB220^™^ on the mesolimbic system using qEEG. In this study, we demonstrate that its intravenous administration reduces or “normalizes” aberrant electrophysiological parameters in reward circuitry. According to our pilot study, one intravenous dose of Synaptamine Complex Variant KB220^™^ significantly normalizes the qEEGs of an alcoholic and heroin abuser with existing abnormalities (i.e., widespread theta and widespread alpha activity, respectively) during protracted abstinence. To determine whether either patient carries putative dopaminergic risk alleles that may predispose them to alcohol or heroin dependence, they genotyped for a number of neurotransmitter reward genes. Among the genes tested were the dopamine transporter (DAT1), the dopamine D4 receptor exon 3 VNTR (DRD4), the DRD2 TaqIA gene (rs1800497), the COMT val158 met gene (rs4680), the monoamine oxidase A upstream VNTR gene (MAOA-uVNTR), and the serotonin transporter-linked polymorphic region (5HTTLPR, loci symbol SLC6A4). All putative risk alleles are unlikely to be carried by all individuals, and this is a case study. In light of previous research and our qEEG studies (parts 1 and 2), we suggest that long-term activation of dopaminergic receptors (i.e., DRD2 receptors) will result in their proliferation, leading to an increase in “ dopamine sensitivity” and happiness, particularly in carriers of the DRD2 A1 allele. A clinical trial using Synaptamine Complex Variant KB220^™^ demonstrated significant reductions in RDS behaviors in > 600 alcoholic patients. As part of this study, we published an expanded oral study on Synaptose Complex KB220Z^™^. In future studies, fMRI and positron emission tomography (PET) scanning will be required to determine the acute and chronic effects of oral KB220^™^ on the number of D2 receptors and their direct interaction with the NAc. Confirmation of these results in large, population-based, case-controlled experiments is necessary. These studies would provide important information that could ultimately lead to significant improvement in recovery for those with RDS and dopamine deficiency as a result of a multiple neurotransmitter signal transduction breakdown in the BRC.

#### Neuronutrient amino-acid therapy protects against RDS: Dopaminergic key to homeostasis and neuroplasticity [78]

An occipital cortex and cerebellum cue activation deficit was observed in Willuhn et al. when habitual cocaine users reduced D2/D3 receptors. In the treatment of psychoactive drugs and behavioral addictions, dopamine agonists maintain dopamine function and prevent relapse. RDS behavior cannot be effectively treated long-term using MAT focused on glutaminergic drugs. Despite the support for short-term use of “dopamine antagonist therapy,” the long-term concept of “dopamine agonist therapy” is proposed based on research. Understanding treatment response and clinical outcomes requires an understanding of neurogenetics and epigenetics. Recovery from drug addiction and non-drug addiction depends on neuro-mechanisms involving “dopamine homeostasis.” Individuals carrying the DRD2 A1 allele (30 – 40 less D2 receptors) may benefit from Neuro-nutrient-Amino Acid Therapy (KB220 variants). Dopamine biosynthesis takes place before L-amino acid decarboxylase is activated in DRD2 A1 carriers. In addition, carriers with amino-acid deficiency (ATPD) exhibit altered decision-making and reward behavior. The Iowa Gambling Task quantified attenuated rewards and reduced decision-making abilities. The question that should be addressed in future research is whether “dopamine agonist therapy” with KB220 variants will improve DRD2 expression, especially in DRD2 A1 allele carriers, and reduce drug and non-drug seeking behaviors through reducing methylation and increasing acetyl groups?.

#### Fifty years in the development of a GDOC (KB220) to balance brain reward circuitry in RDS: A pictorial [79]

Brain reward processing depends on dopamine and other chemical messengers like serotonin, cannabinoids, endorphins, and glutamine. Opiates and opioids are causing a devastating epidemic in the US. Each day, 127 people die due to narcotic overdoses, including both young and old, and heroin overdoses are on the rise. MATs have been approved by the FDA for alcoholism, opiate addiction, and nicotine dependence, but not for abuse of psychostimulants and cannabis. In the short-term, these drugs induce “psychological extinction,” but in the long-term, caution is necessary because they negatively affect dopaminergic function, which is necessary for normal satisfaction. In spite of MATs not being an ideal treatment option, the two institutions that handle alcoholism and drug dependence (NIAAA and NIDA) continue to search for better alternatives. In this article, we examine KB220’s history of development in order to achieve a potential balance of the brain reward system and induce “dopamine homeostasis” by balancing the glutaminergic-dopaminergic system. In addition to providing substantial clinical benefit to RDS victims, this complex may help them recover from iatrogenically induced addiction to unwanted opiates/opioids and other addictive behaviors.

#### Translational and molecular cytoarchitectural genetic guided therapy to induce dopamine homeostatic neuro-signaling in reward deficiency and associated drug and behavioral addiction seeking: A 60 year sojourn the future is now [80]

Globally, behavioral and drug addictions cost society trillions of dollars due to their debilitating effects on individuals. The positive reinforcement of self-medication may be effective, even in the presence of epigenetic negative reinforcement, despite polymorphic DNA reward genes. Chronic abuse of substances and non-substances increases neuronal dopamine release in the NAc, resulting in changes in neural circuits controlling motivational processes, such as arousal, reward, cognition, and stress, while acute use increases neuronal dopamine release. Furthermore, we found significant odds ratios in favor of cases in a large meta-analysis of AUD involving approximately 110,000 cases and 120,000 controls for 8 of the 10 genes and associated alleles (DRD1–4, DAT1, COMT, OPMR, and 5-HTTLR). The GARS test, not a diagnostic but a genetic risk assessment, has shown promise as a tool for early identification in SUD, medical monitoring capabilities, obesity and eating disorder risk, high risk for addiction liability in pain clinics, negative emotionality in chronic cannabis misusers, prediction of clinical outcomes in bariatric patients, profound reduction of prison time for DWI offenders adjudicated to rehabilitation and even in terms of providing genetic risk in children of alcoholics. The development of translational and molecular cytoarchitectural frameworks in relation to genetic and epigenetic insults on reward deficiency and drug abuse, gene therapy neurotransmitters, and exercise molecular mechanisms of pain and anti-reward syndrome has been studied by many researchers (along with a unique measure of dopamine in brain reward circuits). Based on our research, we propose a paradigm shift called “PBM^®^”.

#### Hypothesizing that, a pro-dopamine regulator (KB220X) should optimize, but not hyper-activate the activity of trace amine-associated receptor 1 (TAAR-1) and induce anti-craving of psychostimulants in the long-term [81]

FDA approvals for treating psychostimulant dependence have not been granted for other drugs of abuse such as alcohol, nicotine, and opiates/opioids. This class of stimulant substances is widely accepted to be abused as a result of dopaminergic signaling. Additionally, cocaine has powerful inhibitory effects on the dopamine transporter system, as well as affecting neuronal dopamine release at the NAc. It is understandable that some individuals are more vulnerable to abusing substances of this class. Trace amines activate the TAAR1. Dopamine, serotonin, epinephrine, and histamine have little or no effect on the encoded protein, but beta-phenylethylamine, p-tyramine, octopamine, and tryptamine have a significant effect. There are no introns in this gene. As well as reducing the neurochemical effects of cocaine and amphetamines, TAAR1 agonists also attenuate their addiction and abuse potential. An antagonist of TAAR1 works by blocking dopamine activity in the limbic system, thereby decreasing a hyperdopaminergic state, whereas TAAR1 antagonists work in reverse. In RDS, it is accepted that there is weakened tonic and improved phasic dopamine release, resulting in hypodopaminergic/glutamatergic symptoms. In a number of clinical trials, including neuroimaging studies, the dopamine pro-complex mixture KB220 has been shown to enhance resting state functional connectivity in humans (abstinent heroin addicts), naive rodent models, and regulate theta action in the cingulate gyrus of abstinent psychostimulant abusers. We hypothesize that KB220 may affect resting state functional connectivity, for example, by balancing (optimizing) the effects of TAAR1 on the glutamatergic system so that this system can be optimized. As a result, NAc dopamine will be released at a normal, homeostatic level. An individual’s well-being should be increased by this proposed optimization, not by enhanced activation of TAAR1. If the TAAR1 system is hyper-activated instead of optimized, it will lead to prolonged hypodopaminergic states and, as a result, to enhanced craving for not only psychoactive substances, but also other drug-related and even non-drug related RDS behaviors. Based on the global epidemic of drug and behavioral addictions, this hypothesis will require extensive research.

#### Early intervention of intravenous KB220IV--NAAT improves behavioral outcomes in a residential addiction treatment program: A pilot study [82]

The hypodopaminergic function regulated by reward genes is the culprit in SUDs. An intravenous IV and oral dopaminergic agonist, KB220, was evaluated in the treatment of SUD with the aim of improving dopaminergic function. Using the Chronic Abstinence Symptom Severity (CASS) scale, we found a significant reduction of chronic symptoms. Over the first week and 30-day follow-up period, the combined group (IV and oral) performed significantly better than the oral-only group. For baseline CASS-Revised (CASS-R) variables, 129 subjects were given the combination, and three factors, emotion, somatic, and impaired cognition, with eigenvalues greater than one, were extracted. Paired sample t-tests for pre- and post-treatment scales showed significant declines (p = 0.00001) from pre- to post-treatment: t = 19.1 for emotion, t = 16.1 for somatic, and t = 14.9 for impaired cognition. Two years after KB220IV therapy (at least five IV treatments over seven days) plus orals for 30+ days, 23 subjects remained sober for a total of 21 (91%) months, 19 (82%) without relapse; 19 (82%) were sober at a year, 18 (78%) without relapse; and 21 (91%) were sober two years after treatment, 16 (70%) without relapse. These encouraging results should be interpreted with caution until further research is conducted.

#### Neurogenetics of dopaminergic receptor super-sensitivity in activation of brain reward circuitry and relapse: Proposing “deprivation-amplification relapse therapy” [83]

People who are abstinent for a long time often experience a powerful euphoria, which often leads to a relapse. A biological explanation for this conundrum remains elusive, but we hypothesize that genetic dopaminergic polymorphisms could play a role. Bromocriptine, a dopaminergic agonist, induces stronger activation of brain reward circuitry in individuals who carry the DRD2 A1 allele compared with those who carry the DRD2 A2 allele. This poses another therapeutic conundrum. As dopamine receptor density is significantly lower in carriers of the A1 allele of the DRD2 gene compared to carriers of the A2 allele, they should be less sensitive to dopamine agonists. Bromocriptine, a dopamine D2 agonist, enhances reward sensitivity even with low D2 density. It has also been demonstrated in vitro that D2 receptors proliferate under chronic or long-term therapy with D2 agonists, such as bromocriptine. Several studies have demonstrated that the A1 allele of the DRD2 gene is associated with increased striatal activity of L-amino acid decarboxylase, the final step in dopamine biosynthesis. The amino acid precursor L-tyrosine appears to be used for preferential synthesis of dopamine due to this protective mechanism against low receptor density. A1 carriers appear to have significantly better treatment compliance as a result of receptor proliferation returning to normal levels. In this study, we propose that low D2 receptor density and polymorphisms of the D2 gene are associated with relapses in alcohol dependence, heroin craving, cocaine dependence, methamphetamine abuse, nicotine sensitization, and glucose craving. As a result, we propose a possible physiological explanation for the increased sensitivity seen following intense acute activation of dopaminergic D2 receptors: “denervation super-sensitivity.” The 6-hydroxydopamine rotational model shows an increase in sensitivity to dopamine agonists by 30 to 100x in rats with unilateral neostriatal dopamine depletion. The extent of behavioral super-sensitivity cannot be explained by a simple increase in receptor density, since mild DRD2 proliferation occurs in the striatum (20% - 40%). Therefore, dopamine D2 agonists would target D2 sensitization and attenuate relapse, especially in individuals with the D2 receptor A1 allele. Clinical trials using amino acid neurotransmitter precursors, enkephalinase, and COMT enzyme inhibitors have shown reduced relapse rates in RDS patients. If future translational research reveals that dopamine agonist therapy reduces relapse in RDS, it will support the proposed concept, which we term “deprivation-amplification relapse therapy.” This term couples the mechanism for relapse, which is “deprivation-amplification,” especially in DRD2 A1 allele carriers with natural D2 agonist therapy utilizing amino acid precursors and COMT and enkephalinase inhibition therapy.

#### Neurogenetics and clinical evidence for the putative activation of the brain reward circuitry by a neuroadaptagen: Proposing an addiction candidate gene panel map [84]

NAAT is a neuroadaptagen containing amino-acid neurotransmitter precursors KB220/KB220Z that may enhance reward function in the brain by inhibiting enkephalinase-COMT. A neuroadaptagen that activates the brain reward circuitry is the first known neuroadaptagen. Researchers continue to confirm the numerous clinical effects that ultimately result in significant benefits to addicts, compulsive users, and impulsive users with genetic antecedents for these disorders. It is correct to classify these behaviors as “RDS”. The proposed addiction gene map is a novel approach to understanding the genetic basis of addiction. The effects of oral NAAT on brain reward circuitry activation in SUD victims were studied using qEEG in the US and fMRI in China. Using an fMRI 2×2 design at resting state, NAAT showed activation of the caudate brain region and potential smoothing of heroin-induced putamen (a site for emotionality) connectivity abnormalities. In contrast, if qEEG results published in America show that NAAT increases alpha and low beta, although still awaiting final analysis, NAAT may be shown to improve treatment outcomes.

#### Enhanced functional connectivity and volume between cognitive and reward centers of naïve rodent brain produced by pro-dopaminergic agent KB220Z [85]

The literature supports the existence of dopaminergic reward dysfunction in addictive behaviors. Substance-related disorders are thought to be influenced by alterations in synchronized neural activity between regions of the brain involved in reward and cognitive functions. In this study, KB220Z was shown to significantly enhance reward and cognitive brain connectivity, above placebo, in the rat using a pro-dopaminergic nutraceutical. A number of these are located in the NAc, the anterior cingulate gyrus, the anterior thalamus nucleus, the hippocampus, the prelimbic region, and the infralimbic region. Brain reward circuitry showed significant functional connectivity, increased brain connectivity volume recruitment (possibly neuroplasticity), and dopaminergic functionality. These regions experienced increases in functional connectivity that were not widespread throughout the brain. While these initial findings have been observed in drug naive rodents, this robust, yet selective response suggests clinical relevance for addicts at risk for relapse who demonstrate reduced functional connectivity following prolonged withdrawal. Animal models of addiction will be evaluated in future studies using KB220Z.

#### Pro-dopamine regulator, KB220Z, attenuates hoarding and shopping behavior in a female, diagnosed with SUD and ADHD [86]

Addictive-like behaviors (e.g., hoarding and shopping) may be the result of the cumulative effects of dopaminergic and other neurotransmitter genetic variants as well as elevated stress levels. We, therefore, propose that dopamine homeostasis may be the preferred goal in combating such challenging and unwanted behaviors, when simple dopaminergic activation through potent agonists may not provide any resolution. C.J. is a 38-year-old, single, female, living with her mother. She has a history of substance use disorder as well as ADHD, inattentive type. She had been stable on Bup/Nal combination and amphetamine, dextroamphetamine mixed salts for many years when unexpectedly she lost her job for oversleeping and not calling into work. KB200Z (a pro-dopamine compound) was added to her regimen for complaints of low drive and motivation. After taking this nutraceutical for 4 weeks, she noticed a marked improvement in her mental status and many behaviors. She noted that her shopping and hoarding addictions had appreciably decreased. Furthermore, her lifelong history of terrifying lucid dreams was eliminated. Finally, she felt more in control; her locus of control shifted from external to more internal. The hypothesis is that C.J.’s reported, behavioral, and psychological benefits resulted from the pro-dopamine-regulating effect of KB220Z across the brain reward system. Conclusions, this effect, we surmise, could be the result of a new dopamine balance across C.J.’s brain reward system. Dopamine homeostasis is an effect of KB220Z seen in both animal and human placebo-controlled fMRI experiments.

#### Hypothesizing that a pro-dopaminergic regulator (KB220Z^™^ liquid variant) can induce “dopamine homeostasis” and provide adjunctive detoxification benefits in opiate/opioid dependence [87]

A protocol was developed to be used in treatment centers to initiate detoxification of patients addicted to opiates/opioids (along with some other anti-withdrawal agents). Only three out of 17 subjects received Bup/Nal and KB220Z. For this pilot study, we used KB220Z twice daily before meals along with clonidine, BADs, and other anti-nausea and sleep aids, including gabapentin. Five individuals received KB220Z for six days. Using a higher dose, we repeated the process over a 6-day period, consuming 4 ounces every 6 h. Another 12 patients received the higher dose. In the first two weeks of the study, only 3 people have relapsed on these two protocols, which allowed the remaining 82% to remain on KB220Z. In one subject, 214 days have passed without any additional Bup/Nal. The Clinical Opioid Withdrawal Scale pre- and post-KB220Z is being tested in multiple treatment centers across the US. This hypothesis is currently being tested in multiple treatment centers across the country. A preliminary study agrees with an earlier one even though this is not an acceptable controlled experiment. As KB220Z is used in this detoxification protocol with standard detoxifying agents, we cannot infer its effects. It’s interesting to note that only three out of 17 subjects required Bup/Nal. This method of opiate/opioid detoxification may offer a novel way to eliminate the need for addictive opioids during withdrawal and detox. If further confirmed in larger studies, it may offer a way to eliminate the need for addictive opioids during withdrawal and detoxification. As a result of this paradigm shift, patients at high genetic risk for opioid addiction may no longer need to use powerful and addictive opioids, such as buprenorphine and methadone, as not only detoxifying agents, but also maintenance drugs. Although more research is needed, this pilot could lead to future studies that could reduce addicting opiate/opioid use and mortality amongst Americans of all ages.

#### Improvement of long-term memory access with a pro-dopamine regulator in an elderly male: Are we targeting dopamine tone? [88]

Short-term memory declines with aging, as does long-term memory. Dopamine-related epigenetic insults (e.g., DRD2, DAT1) as well as impaired or reduced mRNA transcription may amplify this effect. Additionally, brain DRD2 is reduced as we age, and aging-induced dementia is associated with an acceleration in DRD2 reduction. Consequently, the authors examined the acute effect of KB220Z on long-term memory performance in a 77-year-old, highly functional male using the Animal Naming Test (ANT). The subject’s follow-up neurology exam revealed an improvement in long-term memory retrieval after he had been taking KB220Z for other reasons. From 2013 to 2016, the patient received several ANTs from his primary and, later, a second neurologist. A non-parametric Wilcoxon-Mann-Whitney test was conducted to test mean differences because the number of ANT observations was small (N = 7 with two groups). The patient scored higher on the ANT after taking KB220Z (p = 0.04762) than when not taking it. A first administration of KB220X raised his scores from the 30^th^ percentile (pre-test) to the 76^th^ percentile, and a second administration of KB220Z raised his scores to the 98^th^ percentile, six months later. A highly functional, elderly male was shown to be significantly more capable of retrieving long-term memory with KB220Z when given acutely. It is recommended that more large, double-blind, randomized controlled studies be conducted.

#### Putative dopamine agonist (KB220Z) attenuates lucid nightmares in PTSD patients: Role of enhanced brain reward functional connectivity and homeostasis redeeming joy [89]

To induce lucid dreams, dreamers have developed training techniques. As well as alleviating nightmares, lucid dreams have been used to induce lucid dreams. Psychiatric diagnoses associated with lucid dreams include PTSD and RDS. Lucid dreams can assume a frightening and unpleasant character in these conditions. PTSD patients with terrifying lucid dreams reported dramatic relief in two cases. The first case study involved a 51-year-old obese woman, diagnosed with PTSD and depression, who attempted suicide and suffered lucid nightmares associated with childhood sexual/physical abuse by her alcoholic father and other family members. In spite of 6 months of treatment with Dialectical Behavioral Therapy and standard pharmaceuticals, including prazosin, clonidine, and Adderall, her vivid “bad dreams” persisted. PTSD patient number two, 39, also suffered from lucid nightmares. After KB220Z was added to the first patient’s medication regimen, the notes reveal changes in the frequency, intensity, and nature of these dreams. Happy dreams extended over a long period of time for the patient for the first time. As an incidental result of administering KB220Z to the second PTSD patient who experienced lucid nightmares, he reported dreams containing laughter and joy. In human and rodent experiments, KB220Z is shown to enhance dopamine homeostasis and functional connectivity of reward circuits in the brain. Double-blinded, large-scale studies are needed to gain a deeper understanding of their effects.

#### rsfMRI effects of KB220Z^™^ on neural pathways in reward circuitry of abstinent genotyped heroin addicts [90]

As dopaminergic function is reduced, cocaine use and even non-substance-related addictive behavior increases. In a recent PET study by Volkow’s et al., chronic cocaine exposure was associated with decreased D2/D3 receptors and decreased activation of cues in the occipital cortex and cerebellum. In order to prevent relapses in psychoactive drug and behavioral addictions, treatment strategies that preserve dopamine function are an interesting approach. In order to test how KB220Z^™^ affects reward circuitry, we studied 10 heroin addicts who had been abstinent for an average of 16.9 months. In a randomized placebo-controlled crossover study of KB220Z, five subjects completed a triple-blind experiment in which the subject, the person administering the treatment, and the person evaluating the response to treatment were all blind to the treatment that any particular subject was receiving. The GARSDx^™^ test was also used to genotype nine subjects. After acute administration of KB220Z for one hour, we found an increase in BOLD activation in caudate-accumbens-dopaminergic pathways compared to placebo. Additionally, abstinent heroin addicts were found to have lower levels of resting-state activity in the putamen after taking KB220Z. Three brain regions of interest were significantly activated by KB220Z from resting state in this pilot study of abstinent heroin-dependent subjects (p=0.05) in the second phase. In a putative network including the dorsal anterior cingulate, the medial frontal gyrus, the NAc, the posterior cingulate, and the occipital cortex, functional connectivity was observed. A direct or indirect dopaminergic interaction with KB220Z may have an anti-craving/anti-relapse effect in addiction, based on these results and other qEEG studies. We caution definitive interpretation of these preliminary results due to the small sample size. Further research as well as ongoing rodent and human studies are required to confirm these preliminary findings.

#### Overcoming qEEG abnormalities and reward gene deficits during protracted abstinence in male psychostimulant and polydrug abusers utilizing putative dopamine D2 agonist therapy: Part 2 [91]

The DRD2 gene A1 allele is associated with “dopamine resistance” in both food- and drug-addicted individuals. Evidence suggests that a natural, nonaddictive, safe, putative D2 agonist could play an important role in helping individuals with RDS, including those who are addicted to psychoactive chemicals, recover. In a randomized, triple-blind, placebo-controlled, crossover study, qEEG imaging indicated an increase in alpha waves and low beta waves in the parietal brain region following oral Synaptose Complex KB220Z^™^. Following week one and week two of analyses, significant differences between placebo and Synaptose Complex KB220Z^™^ were observed in the frontal regions (p = 0.03). Prefrontal cortex involvement in the response to a D2 agonist (Synaptose Complex KB220Z^™^) is demonstrated here for the first time, especially in subjects with the dopamine D2 A1 allele. Additionally, we tested 3 polydrug abusers who carried the DRD2 A1 allele undergoing protracted abstinence to support this finding. qEEG patterns were significantly different between placebo and Synaptose Complex KB220Z^™^ groups when placebo was administered as part of a study. When the brain’s dysregulated electrical activity was stimulated, these addicts responded positively to Synaptose Complex KB220Z^™^. Results indicate a phase change from a low-amplitude or low-power state to a more regulated state when 6.169 mV is increased across the prefrontal cortex. Among the subjects in the first experiment, 50% carried the DRD2 A1 allele, whereas 100% carried the risk allele. According to the proposed addiction risk score, 72% of these 14 subjects had moderate-to-severe addiction risk. Three additional polydrug abusers had the DRD2 A1 allele. The results of this seminal study could have a major impact on people who are addicted to stimulants and polydrugs and suffer from genetically induced dopamine deficiencies. Based on this small sample size, we are proposing that with necessary large populations supporting these initial results, and possibly even additional candidate genes and single nucleotide polymorphisms, it is possible to eventually classify severity by genotype and risk alleles, as well as provide a natural, safe, nonaddictive dopaminergic receptor agonist that could upregulate rather than downregulate dopaminergic receptors, preferably the D2 subtype.

#### KB220Z^™^ a pro-dopamine regulator associated with the protracted, alleviation of terrifying lucid dreams. Can we infer neuroplasticity-induced changes in the reward circuit? [92]

Several studies conducted by our laboratory suggest that lucid dreams are associated with psychiatric conditions, including ADHD and RDS. Observations have shown that such lucid dreams can be unpleasant and often frightening. Our study presents four cases of patients with ADHD/PTSD and/or opiate/opioid dependency experiencing dramatic and persistent relief from terrifying, lucid dreams. Despite the patients’ inability to recall such dreams for at least 10 months, the amelioration could well be permanent, since the nutraceutical had been stopped for at least 12 months without any recurrence or remembrance of the dreams. A 47-year-old married male required continued Bup/Nal (Suboxone) treatment in the first case. In the second case, the subject was a 32-year-old female with ADHD. A 38-year-old male with substance use disorder and ADHD is the third case. A 50-year-old female with alcohol abuse, ADHD, and PTSD was the fourth case. In order to attempt to understand the possibility of neuroplasticity, we evaluated the effect of KB220Z in non-opioid-addicted rats utilizing fMRI methodology. There may be some differences between rat and human brain functional connectivity, which makes it difficult to make a definitive claim, but we do gain some interesting insights from it. With the exception of the prefrontal cortex, connectivity volume was enhanced following seeding of the dorsal hippocampus. In lucid human dreaming, however, the latter region is rarely activated, when the dreamer reports that they had the feeling, they were dreaming. After taking KB220Z, the four patients reported gradual improvement in their long-term, terrifying, lucid dreams. A self-initiated cessation of KB220Z use resulted in persistent amelioration of these dreams for up to 12 months. As a result of these particular cases, it is possible that KB200Z increases both dopamine stability as well as functional connectivity between reward circuitry networks in rodents and humans. The increase in connectivity volume in rodents may be analogous to those associated with lucid dreaming and rapid eye movement sleep in humans. More intensive investigations involving large-scale, double-blinded studies are needed to determine whether the complex induces long-term, neuroplastic changes.

#### Hypothesizing repetitive paraphilia behavior of a medication refractive Tourette’s syndrome patient having rapid clinical attenuation with KB220Z-NAAT [93]

Many patients with multiple behaviors such as drug abuse and food addiction, as well as pathological repetitive behaviors like gambling, self-mutilation, and paraphilia, may not be properly diagnosed. The present case describes a male who presented many of these seemingly diverse behaviors but was ultimately diagnosed with RDS by his attending physician. After two weeks of using the dopamine agonist, ropinirole, sexual behavior improved, but tolerance set in and treatment was discontinued, especially when an insurance infraction occurred. We examine how ropinirole can cause adenylate cyclase receptor super-sensitivity and tolerance, which is a characteristic of neurotransmitter crosstalk, in this article. It was determined that KB220Z-NAAT activated dopaminergic pathways in both the prefrontal cortex cingulate gyrus (relapse loci) and the ventral tegmental area-caucadate-accumbens-putamen (craving and emotion loci) rapidly (post one hour). The repetitive paraphilia disappeared a week after using the treatment. A positive effect of KB220Z is enhanced focus, which lasts even after the patient stops taking it (such as altruistic thoughts). However, large cohorts are needed to study these benefits in greater depth. Despite focusing on a rapid response rather than long-term benefits previously associated with NAAT, this report is somewhat encouraging, and longer-term follow-up and larger placebo-controlled studies would be required before any definitive conclusions could be drawn.

#### Low-resolution electromagnetic tomography of changed brain function provoked by pro-dopamine regulator (KB220Z) in one adult ADHD case [94]

The symptoms of ADHD often persist into adulthood. Studies have shown that affected individuals suffer from lower baseline levels of dopamine in their brains, which may increase their risk of SUD. The purpose of this study was to determine whether there was potential for novel management of adult ADHD using KB200Z, a non-addictive GDOC. A 72-year-old male with ADHD was evaluated at baseline and one hour after administration of KB220Z using low-resolution electromagnetic tomography. Working memory, with KB220Z, resulted in higher z-scores for each frequency band, in the anterior, dorsal, and posterior cingulate regions, as well as the right dorsolateral prefrontal cortex. It is consistent with other human and animal neuroimaging studies that have shown increased connectivity volumes in reward circuitry, suggesting a new approach to ADHD treatment. These results need to be confirmed by larger randomized trials.

#### “Pro-dopamine regulation (KB220Z^™^)” as a long-term therapeutic modality to overcome reduced resting state dopamine tone in opiate/opioid epidemic in America [95]

As an example, carriers of the DRD2 A1 allele have a tendency to prefer amino-acid therapy, which is associated with relapse, morality, and hospitalization. As a front-line tactic to overcome the current American opiate/opioid epidemic, it seems intuitive to incorporate modalities to restore hypodopaminergic balance and or restore hypodopaminergic relapse, saving millions of people from death and unwanted addiction. Our efforts to fight addiction to narcotics with narcotics will fail if we follow the prim road. Global interest can also be derived from this lesson.

#### RDS: Attentional/arousal subtypes, limitations of current diagnostic nosology, and future research [96]

Narcolepsy and hypersomnia are associated with ADHD. The pathophysiology of ADHD may be similar to that of addictions (RDS). A narrow nosological framework may prevent such connections from being recognized. Additionally, we hypothesize that narcolepsy develops in people with ADHD/RDS and further damages their dopaminergic reward system. This study will test these hypotheses using a combination of genetic screening, neuroimaging, and pharmacological interventions in subjects with ADHD, narcolepsy, or both. Combined ADHD-narcolepsy may have a common pathophysiology, as well as an additional compromise to the reward system. The potential dopaminergic compound KB220Z^™^ may be beneficial to those with ADHD if the evidence supports the hypothesis that narcolepsy and RDS share a pathophysiology.

#### Can the chronic administration of the combination of Bup/Nal block dopaminergic activity causing anti-reward and relapse potential? [97]

Overdose deaths, infectious disease transmission, increased health care costs, public disorder, and crime are all associated with opioid addiction. Despite the fact that community-based addiction treatment programs continue to reduce the harms caused by opiate addiction with narcotic substitution therapy such as methadone maintenance, a substance that blocks both opiate-type receptors (mu, delta, etc.) as well as provides an agonistic effect remains needed; therefore, a combination of mu receptor agonist therapy and narcotic antagonism was developed. Over the last three decades, extensive research has provided patients with addiction disorders with more treatment options. Treatment for opioid dependence can be achieved by completing a brief specialty-training course in buprenorphine and Bup/Nal (Subutex, Suboxone). Clinical studies have shown that buprenorphine maintenance is as effective as methadone maintenance in reducing illicit opioid use and retaining patients in treatment. Subutex or Suboxone may be toxic in the long run, so it’s important to consider their benefits. In spite of only partial opiate agonist effects, chronic blockade of opiate receptors may cause anti-reward and relapse potential. As of yet, there are no direct comparisons available, but the scientific literature does mention buprenorphine’s toxicity. As we consider our cautionary note in this commentary, we are aware that, to date, these are the only tools available to us, and until the real magic bullet comes along, we will have to put up with what we have. Most importantly, this commentary should at least encourage the development of thoughtful new strategies for preventing relapses by targeting specific brain regions.

#### Enkephalinase inhibition: Regulation of ethanol intake in genetically predisposed mice [98]

This is the first report on altered alcohol intake in mice with an innate deficiency in brain enkephalin and genetic predisposition to alcohol preference. Hydrocinnamic acid and D-phenylalanine, carboxypeptidase (enkephalinase) inhibitors, have significantly reduced volitional and forced intakes of ethanol, respectively. Our hypothesis is that altering endogenous brain opioid peptides can reduce excessive alcohol intake by inhibiting enkephalinase.

#### L-DOPA: Effect on ethanol narcosis and brain biogenic amines in mice [99]

The narcosis induced by ethanol is augmented in mice by L-DOPA1. L-DOPA administration is associated with a marked increase in brain dopamine, based on the analysis of brain biogenic amines. L-DOPA-induced ethanol narcosis is further enhanced by dopamine here.

#### Improvement of inpatient treatment of the alcoholic as a function of neurotransmitter restoration: A pilot study [100]

The nutritional supplement SAAVE was evaluated for its ability to facilitate improvement in an inpatient alcohol and drug rehabilitation center over a 30-day period. In alcoholics, SAAVE elevates levels of enkephalin(s), serotonin, catecholamines, and GABA, which are functionally deficient. A total of 22 patients were studied. Comparing SAAVE patients to control group patients, (a) the BUD score (building up to drink) was lower, 1 vs 2. (b) PRN BZDs were not required, 0% vs 94%. (c) tremors stopped after 72 h, as opposed to 96 h; and (d) 24% of control group patients did not have severe depression. Preliminary studies suggest that SAAVE facilitates a more positive response to behavioral therapy by aiding the patient’s physical adjustment to a detoxified state.

#### Enkephalinase inhibition and precursor amino acid loading improves inpatient treatment of alcohol and polydrug abusers: Double-blind placebo-controlled study of the nutritional adjunct SAAVE [101]

In order to evaluate the role of neurotransmitters in facilitating recovery and adjustment to a detoxified, sober state, we studied the effects of the amino acid and vitamin mixture SAAVE on inpatient, chemically dependent subjects. In SAAVE, amino acids are incorporated that are precursors to neurotransmitters and neuromodulators that are believed to play a role in alcohol and drug cravings. SAAVE patients significantly reduced their stress response, as measured by their skin conductance level, in a double-blind, placebo-controlled, randomized study of 62 alcoholics and polydrug abusers. They also significantly improved their physical scores and BESS scores (behavioral, emotional, social, and spiritual). Comparison of SAAVE and placebo groups showed a six-fold decrease in AMA rates after detoxification. By measuring the BESS Score, SAAVE facilitated the rate of recovery and helped patients respond more fully to the behavioral goals of the program. A practitioner can potentially restore neurochemical changes associated with alcoholism and drug abuse by using SAAVE to inhibit enkephalinase and load precursor amino acids in the acute inpatient treatment environment. Compulsive disorders are underpinned by neurobiological mechanisms that are clinically relevant.

#### Reduction of both drug hunger and withdrawal against advice rate of cocaine abusers in a 30-day inpatient treatment program by the neuronutrient Tropamine [102]

There were three groups in a study involving 54 cocaine-dependent mixed gender patients who were treated in a residential setting for 30 days: administration of KB220 specific formula for psychostimulant abuse called Tropamine (T); administration of KB220 specific formula for alcohol abuse called SAAVE; and no supplement. According to the results, the AMA rates were as follows: [C] 37.5%, [SAAVE] 26.6%, and [Tropamine] 4.2%. As well as reducing drug hunger, Tropamine also decreased AMA rates. Precision medication therapy is now underway. Despite the fact that the basic formula relating to pro-dopamine regulation is tantamount to neurotransmitter dysfunction, specific formulations regarding the drug of choice appear more prudent as treatment options.

#### Neurodynamics of relapse prevention: A neuro-nutrient approach to outpatient DUI offenders [103]

The administration of SAAVE for alcoholics and Tropamine for cocaine dependents in a 10-week outpatient treatment program resulted in reduced relapse rates and enhanced recovery. SAAVE was administered to alcoholics for ten months and led to a 73% recovery rate (with 26% relapse). Also, 53% of cocaine addicts recovered after receiving Tropamine (47% relapsed). Between 87 and 93% of people who have relapsed from alcoholism or cocaine dependence. It is certain that this finding fits into the current drug court dogma pointing to the importance of inducing dopamine homeostasis to facilitate long-term recovery.

#### Neuro-nutrient effects on weight loss in carbohydrate bingers; an open clinical trial [104]

Over a 90-day program, 27 carbohydrate bingers were treated with a variant of KB220 called PhenCal 103 in an outpatient bariatric clinic, 16 of whom received PhenCal 103 and 11 of whom did not. Based on the results, an average of 26.96 pounds was lost as a result of PCAL-103. Comparatively, 10.2 were found in the control group. PCAL-103 had an 18.2% relapse rate compared to 81.8% in the control group. As a subclassification of RDS, Obesity is an important component of this study.

#### Enhancement of attention processing by Kantroll^™^ in healthy humans: A pilot study [105]

It is the first report in humans showing that Kantroll^™^ (KB220) consumption daily produces performance-related event-related potentials (ERPs). In normal young adult volunteers, two computerized visual attention tasks, the Spatial Orientation Task and the Continuous Performance Task, were used to generate cognitive ERPs. Each subject was used as a control for testing before and after ingestion of amino acids for 28 – 30 days. Following KB220, both P300 components of the ERPs showed statistically significant amplitude enhancements, as well as improvements in cognitive processing times. Researchers observed in this study an increase in neurophysiologic function in normal controls, which is consistent with the facilitation of recovery of individuals with RDS (i.e., substance use disorder, ADHD, carbohydrate bingeing) after taking the amino acid supplement, KB220. Further placebo-controlled, double-blind studies are needed to confirm and extend these findings.

#### NeuRecover-SATM in the treatment of cocaine withdrawal and craving: A pilot study [106]

An experiment involving KB220 variants similar to Tropamine was performed on 12 severe cocaine-dependent patients in a state psychiatric hospital, with eight patients taking KB220 variants and four taking placebos. A significant decrease in cocaine craving was found in the KB220 group compared to the placebo group. Although there were only a few people in this study, it shows that dopamine plays a role in cocaine cravings.

#### Amino-acid precursor and enkephalinase inhibition therapy: Evidence for effectiveness in treatment of “RDS” with particular emphasis on eating disorders [107]

During nine months and three years after treatment with amino-acid precursor and enkephalinase inhibition therapy, six randomly selected females with eating disorders (three of whom were chemically dependent) were contacted. Despite one relapse at six months, all six reported an initial benefit. Additionally, an extended number of 100 former eating disorder patients treated with amino-acid precursors and enkephalinase inhibition therapy reported significant improvements in mood and substance cravings in 98% of the cases. It appears that the consistency of results and the number of subjects suggest that food addiction is similar to drug seeking behavior, even though the study was not randomized, or placebo controlled.

#### Gene\Narcotic Attenuation Program attenuates SUD, a clinical subtype of RDS [108]

The effects of a putative activator of brain reward circuitry were assessed over the course of a one-year prospective comprehensive outpatient program. A SUD patient was treated with Haveos (Synaptamine^™^) (KB220) as part of the Gene Narcotic Attenuation Program. Seventy-six patients with serious SUDs (45 females and 31 males; mean age, 33 years; standard deviation, 7.0) participated in the study. From the time of entry to the end of the study, self-reported craving decreased after excluding 15 patients who dropped out early. In addition to the desire (visual analog scale, where 0 represents no desire and 5 represents the strongest desire), 61 compliant patients showed that their craving decreased significantly (p < 0.001). After 1 year of treatment, the building up to relapse scores (each with a summary value) improved similarly; the mean score decreases were significant for stress (t = 3.3; p = 0.002), depression (t = 4.0; p < 0.001), anger (t = 4.4; p < 0.001), anxiety (t = 4.5, p < 0.001), drug craving (t = 5.4, p < 0.001), and summary building up to relapse (t = 4.1; p < 0.001). Recovery score measures of energy level (t = 8.4; p = 0.001) and ability to refrain from drug-seeking behavior (t = 7.4; p = 0.001) also showed significant increases. A significant difference was observed between alcoholics and psychostimulant users in terms of dropout rates (5 out of 57 versus 10 out of 15). It is important to conduct rigorous double-blind studies before interpreting these results, even though they are significant.

#### Narcotic antagonists in drug dependence: Pilot study showing enhancement of compliance with SYN-10, amino-acid precursors and enkephalinase inhibition therapy [109]

The hypothesis was tested to determine whether combining a narcotic antagonist and amino acid therapy could boost compliance in methadone patients rapidly detoxified with the narcotic antagonist Trexan (Dupont, Delaware) by promoting neuronal dopamine release using enkephalinase inhibitors (D-phenylalanine) and neurotransmitter precursors (L-amino-acids). Accordingly, Thanos et al., the DRD2 gene was delivered to the NAc via an adenoviral vector and increased DRD2. The ethanol preference rates recovered as the DRD2 returned to baseline levels after a significant reduction in ethanol preference (43%) and alcohol intake (64%) was observed. The overexpression of DRD2 also produced significant reductions in alcohol preference (16%) and alcohol consumption (75%) in rats that did not prefer ethanol. Additionally, this study suggests that high levels of DRD2 may protect against alcohol abuse. Numerous studies have also linked the DRD2 A1 allele to heroin addiction. Furthermore, polymorphisms in other dopaminergic receptor genes have also been associated with opioid dependence. For example, Kotler et al. showed that the 7-repeat allele of the DRD4 receptor is significantly overrepresented in the opioid-dependent cohort and confers a relative risk of 2.46. This has been confirmed by Li et al. The 5 and 7 repeat alleles of the DRD4 receptor were detected in the Han Chinese heroin addicts in both cases and controls. Similarly, Duaux et al. identified homozygous DRD3-Bal 1 alleles to be associated with heroin abuse in French addicts. A study from NIAAA provided evidence which strongly suggests that DRD2 is a susceptibility gene for substance abusers across multiple populations. Moreover, there are a number of studies utilizing amino-acid and enkephalinase inhibition therapy showing reduction of alcohol, opiate, cocaine and sugar craving behavior in human trials. Many treatment centers across the US, Canada, as well as many countries on a worldwide basis have become interested in a rapid method to detox heroin or methadone addicts using Trexan in the past decade. In using the combination of Trexan and amino-acids, results were dramatic in terms of significantly enhancing compliance to continue taking Trexan. The average number of days of compliance calculated on 1000 patients, without amino-acid therapy, using this rapid detoxification method is only 37 days. In contrast, the 12 subjects tested, receiving both the Trexan and amino-acid therapy was relapse-free or reported taking the combination for an average of 262 days (p < 0.0001F). Thus, coupling amino-acid therapy and enkephalinase inhibition while blocking the delta-receptors with a pure narcotic antagonist may be quite promising as a novel method to induce rapid detox in chronic methadone patients. This may also have important ramifications in the treatment of both opiate and alcohol-dependent individuals, especially as a relapse prevention tool. It may also be interesting to further test this hypothesis with the sublingual combination of the partial opiate mu receptor agonist buprenorphine.

#### A short-term pilot open label study to evaluate efficacy and safety of LG839, a customized DNA directed nutraceutical in obesity: Exploring nutrigenomics [110]

Using DNA and a customized formulation, a first PBM^®^ experiment indicates that high consumption of alcohol or carbohydrates (carbohydrate bingeing) stimulates the brain’s use of and production of dopamine. Having low dopaminergic activity in the reward center of the brain contributes to obesity. In order to categorize biological and genetic influences upon behavior, this syndrome has been named RDS. Behavioral modifications are needed to adequately treat obese patients at the same time as RDS is addressed. An observational study involving 24 participants documented 15 categories of benefits from using an early form of KB220 called GenoTrim, which is a NAAT formulation customized to each individual’s DNA. Statistics from the survey indicated that stress reduction improves sleep, energy, focus and performance, while reducing appetite, losing unwanted weight, decreasing body inches, and improving well-being. Pro-dopamine regulation has the potential to provide other benefits, such as a better recovery process from overeating, by potentially improving dopaminergic function.

#### Synaptamine^™^ (SG8839), an amino acid enkephalinase inhibition nutraceutical improves recovery of alcoholics, a subtype of RDS [111]

A nutraceutical for improving emotional and behavioral symptoms called Synaptamine (a variant of KB220) helped 600 recovering alcoholics lower their emotional and behavioral symptoms in an open clinical study. There were significant reductions in cravings, depression, anxiety, anger, fatigue, lack of energy, and crisis. In particular, cravings, depression, anxiety, anger, fatigue, and lack of energy were reduced in comparison to pre and post administration scores. As shown in other related studies, oral dosing is potentially more cost-effective than IV therapy for a relatively small number of patients, but this study does support the important role of dopamine regulation in alcoholism.

#### Chromium picolinate (CrP), a putative anti-obesity nutrient induces changes in body composition as function of the Taq1 DRD2 gene [112]

With 122 obese subjects tested for the Taq1 DRD2 gene, the role of CrP, a major component of KB220, was compared with placebo. The DRD2 A1 or A2 gene was genotyped in all 122 subjects. A placebo group (62) and a CrP group (60) were created from this cohort. In this study, there was a significant weight loss and other change in body composition in DRD2 A2 genotype carriers. In contrast, the genotypes A1/A1 and A1/A2 were not significant. Hence, CrP’s differential therapeutic effect on weight loss and body fat changes is associated with the dopaminergic system, specifically D2 receptor density. DRD2 A1 carriers ingested carbohydrates and fat, masking the benefits of CrP. It is one of the first experiments to incorporate pharmacogenetic testing as a nutrigenomic approach to obesity relapse prevention, underscoring the importance of gene polymorphisms and overeating.

#### LG839: Anti-obesity effects and polymorphic gene correlates of RDS [113]

An experimental nutraceutical based on DNA customization was systematically assessed for its potential weight management benefits. During D.I.E.T., 1058 subjects with polymorphic outcomes were genotyped and administered an LG839 variant. The chi-square analysis was performed on 27 obese Dutch subjects who had the same DNA pattern for four of the five genes tested as the entire data set. We conducted simple t tests comparing weight management parameters before and after treatment with LG839 for 80 days. Significant results were observed for weight loss, sugar craving reduction, appetite suppression, snack reduction, reduction of late-night eating (all p < 0.01), increased perception of overeating, enhanced quality of sleep, increased happiness (all p < 0.05), and increased energy (p < 0.001). Polymorphic correlates were obtained for a number of genes (LEP, PPAR-gamma2, MTHFR, 5-HT2A, and DRD2 genes) with positive clinical parameters tested in this study. Of all the outcomes and gene polymorphisms, only the DRD2 gene polymorphism (A1 allele) had a significant Pearson correlation with days on treatment (r = 0.42, p = 0.045). The results of our study suggest that DNA-directed targeting of certain regulator genes, along with customized nutraceutical interventions, could provide a unique framework for overcoming obesity if they are verified in additional rigorous, controlled studies.

#### DRD2 Taq A1 allele predicts treatment compliance of LG839 in a subset analysis of pilot study in the Netherlands [114]

DNA-individualized customized nutraceuticals could be produced by genotyping certain known candidate genes in order to counteract various genetic traits that may contribute to body composition. An analysis was conducted on twenty-one subjects for DRD2, methylenetetrahydrofolate reductases, serotonin receptors (5-HT2as), and the leptin gene (OB). In order to develop the nutraceutical LG839 [DL-phenylalanine, chromium, L-tyrosine, other select amino acids and adaptogens in KB220] that combats obesity with a special focus on body composition (i.e., BMI), these five genes were systematically evaluated as potential critical biological targets. A 41-day trial revealed a trend in weight loss where 71.4% of subjects lost weight. In this experiment, the significance of nutrigenomics in treating obesity has been replicated for a third time.

#### Putative targeting of DRD2 function in RDS by Synaptamine Complex^™^ Variant (KB220): Clinical trial showing anti -anxiety effects [115]

Dopamine is known as the “anti-stress molecule” in the brain. A randomized, double-blind placebo-controlled study investigated the anti-anxiety effects of Synaptamine Complex (previous version of KB220), a pro-dopamine regulator, on alcoholics and polydrug abusers in an inpatient chemical dependency treatment program. Compared to patients who received the placebo, 62 patients who received Synaptamine Complex had significantly reduced stress levels. As a function of time and treatment, as well as their interaction, two-factor measures analyses of variance revealed significant differences. Although these findings provide support for potentially overcoming hypodopamergia due to stress, they do not provide mechanistic evidence regarding the role of Pro-dopamine regulation and its impact on Corticotrophin Releasing Factor. In spite of this, stress can reduce endogenous opioid peptides in the pituitary and striatum while increasing plasma cortisol levels.

#### Targeting noradrenergic and dopaminergic mechanistic sites, hormonal deficiency repletion therapy and exercise: A case report [116]

Diethypropion (Tenuate^®^), a hormonal replacement therapy, the Rainbow Diet^®^, and light exercise were all used in conjunction with Synaptamine Complex (KB220) treatment to obtain sustained weight loss. One year after surgery, the 58-year-old patient’s body mass index decreased from 32 to 25.4. A DEXA scan showed a reduction in his body fat percentage from 36.91% to 17.8%. Known weight loss products work in synergy with this procedure. Induction of putative dopamine homeostasis is therefore necessary to address obesity, which is a multifactorial medical problem.

#### Neurotransmitter-precursor-supplement intervention for detoxified heroin addicts [117]

In this study, tyrosine, lecithin, L-glutamine, and L-5-hydroxytryptophan were administered combined to detoxified heroin addicts to study withdrawal syndromes and mental symptoms. 83 heroin addicts were recruited from a detoxification treatment center in Wuhan, China, for the cluster-randomized placebo-controlled trial. Tyrosine, lecithin, L-glutamine, and L-5-hydroxytryptophan were administered to patients in the intervention group (n = 41) and placebos to patients in the control group (n = 42). Pre- and post-intervention mood states were monitored as well as sleep status and withdrawal symptoms throughout the study. As compared to the control group, participants in the intervention group showed significant improvement in insomnia and withdrawal scores over time. The intervention group showed greater reductions in tension-anxiety, depression-dejection, anger-hostility, fatigue-inertia, and total mood disturbance at day 6 than the control group (all p < 0.05). Patients recovering from heroin addiction may find the neurotransmitter precursor supplement intervention effective in alleviating withdrawal and mood symptoms.

#### Introducing “PAM^®^” as an adjunctive genetic guided therapy for abusable drugs in America [118]

Previously KB220 variants in human trials demonstrated clinical benefit: (a) Significantly reduced relapse rates and enhanced recovery outcomes in 73% of alcoholics and 53% of cocaine-dependent patients, in a ten-month DUI-out-patient study; (b) A triple-blind, placebo-controlled, crossover study of abstinent psychostimulant use disorder patients found regulation of qEEG widespread theta, increased alpha and low beta, and reduced high beta activity in the parietal brain region; (c) A significant difference between placebo and KB220 in cocaine abusers regarding AMA rate (over the first five days) was 37.5% compared to only 4.2% in a 30-day hospital program; and (d) Naive rats significantly increased resting-state functional connectivity volume across various brain regions (neuroplasticity) in a triple-blind, placebo-controlled, crossover study.

In people with genetically induced dopamine deficiency, GARS may provide valuable information. It may be possible to improve the recovery of individuals with, for example, psychostimulant and polydrug addiction problems by using precision KB220 as a treatment adjunct to putatively induce dopamine regulation. Our knowledge of how 85 billion neurons in the human brain are interconnected is at a pioneering stage because of the complexity of RDS behaviors, especially drug-seeking behaviors. In the current arena of neuroimaging and genetic interface, this reductionist view, as presented herein, must be considered as an integral piece of the puzzle.

#### NIDA-Drug Addiction Treatment Outcome Study relapse as a function of spirituality/religiosity [119]

Durkheim first connected religion/spirituality to deviance, such as substance abuse, by defining socially expected behaviors as norms. According to him, deviance is largely caused by their absence (called anomie) and spirituality reduces deviance by preserving social norms and bonds. Symptoms of RDS may also result in deviance, and as a result, we wondered if stronger faith in spirituality and religion would lower relapse. We examined post hoc relapse rates among 2,947 clients broken down by five spirituality measures based on the NIDA- Drug Addiction Treatment Outcome Study data. The main findings suggest that people with low spirituality are more likely to relapse, whereas those with high spirituality are more likely to recover. Crack use is the only exception to this. We found significant differences in terms of cocaine, heroin, alcohol, and marijuana relapse as a function of strength of religious beliefs (χ^2^ = 27.190, p < 0.0005; logistic regression = 17.31, p < 0.0005); frequency of watching religious programs (χ^2^ = 19.02, p = 0.002; logistic regression = ns); and frequency of meditation/prayer (χ^2^ = 11.33, p = 0.045; logistic regression = 9.650, p = 0.002). There was a 7% to 21% difference in alcohol, cocaine, heroin, and marijuana use between spiritual participants and non-spiritual participants across the five measures of spirituality. Religion had no significant effect on crack use, however, for crack users who did not report religion as important. Attending weekly religious services is the best indicator of remission and spirituality, which reflects Durkheim’s social bond theory by involving the most social interaction and social bonding. Remission from abused drugs except crack is directly associated with stronger spiritual/religious beliefs and practices. Regular spiritual practice, particularly attendance at the religious services of their choice on a weekly basis, has the same effect on clients who abuse drugs as having a sponsor. In drug treatment programs, spirituality plays a critical role in creating social bonds and plays a clinically significant role. An appropriate approach may be biphasic, including short-term blockade followed by long-term dopaminergic upregulation, as described in this report. Treatment would entail enhancing brain reward connectivity volume, targeting reward deficiency, and attending self-help groups if stress-like anti-reward symptoms are present. Following KB220 variants, we found significantly lower relapse rates in alcohol, opioid, cocaine, and food dependent patients.

#### Weight gain is associated with reduced striatal response to palatable food [120]

Obese versus lean humans have fewer striatal D2 receptors, which is consistent with the theory that individuals with hypofunctioning reward circuits overeat in order to compensate for a reward deficit. Genetically susceptible individuals with reduced signaling of dopamine-based reward circuitry are predicted to gain weight if their striatal response to food intake is low. Overeating, however, may lead to diminished striatal responsiveness by downregulating D2 receptors, reducing D2 sensitivity, and decreasing reward sensitivity. By using repeated-measures functional magnetic resonance imaging, we investigated whether overeating reduces the response of the striatum to palatable food intake in humans. In women who gained weight over a six-month period, the striatal response to palatable food consumption was reduced compared to women who remained weight stable. Based on these results, it appears that low sensitivity of reward circuitry increases risk for overeating, which may further attenuate reward circuitry responsiveness.

## Policy

### A systematic, intensive statistical investigation of data from the CARD for compliance and illicit opioid abstinence in substance addiction treatment with Bup/Nal [121]

Current treatment options for OUD include Bup/Nal, a combination partial mu receptor agonist and delta mu antagonist. Literature review, however, found very few studies that examined compliance and abstinence based on urine drug tests. A large cohort of Bup/Nal patients attending chemical-dependency programs in eastern US in 2010 and 2011 was analyzed statistically using data from the CARD. Part 1: Bup/Nal was present in 93.4% of first (n = 1,282; p < 0.0001) and 92.4% of last (n = 1,268; p < 0.0001) urine samples. Concomitantly, unreported illicit drugs were present in 47.7% (n = 655, p = 0.0261) of samples. Compliance with the Bup/Nal prescription was associated with higher rates of abstinence during treatment (p = 0.0012; odds ratio = 1.69, 95% CI 1.210, 2.354). Analysis of all samples collected in 2011 shows significant improvements in compliance (p = 2.2 × 10^−16^) and abstinence (p = 2.2 × 10^−16^). Although significant use of illicit opioids is present during treatment with Bup/Bal, improvements in abstinence and high compliance during maintenance-assisted therapy programs may alleviate fears of diversion. Covariates important to further study include the treatment modality, location, and year of sampling. Future studies should consider long-term antireward effects from Bup/Nal use.

### Systematic evaluation of “compliance” to prescribed treatment medications and “abstinence” from psychoactive drug abuse in chemical dependence programs: Data from the CARD [122]

In six eastern states of the country, this is the first comprehensive analysis of urine drug testing to determine whether patients were compliant with their treatment medications and abstinent from drug abuse. Post-hoc retrospective observational study using CARD data on 10,570 patients was conducted, filtered to include 2,919 patients prescribed at least one treatment medication in 2010 and 2011. For many, compliance with treatments medications and abstinence from drugs of abuse supported the effectiveness of treatment. Compliant patients abused opioids, cannabinoids, and ethanol less than non-compliant patients during treatment, but BZDs more than non-compliant patients. During treatment, 17% of non-abstinent patients used BZDs, 15% used opiates, and 10% used cocaine. In residential treatment facilities, compliance rates were significantly higher than in non-residential treatment facilities. There was 67.2% compliance with every medication prescribed both at the first and last urine samples (no difference based on level of care). Furthermore, 39.2% of the patients (n = 1143; p = 0.001) were abstinent in both their first and last urine samples. Furthermore, 13.3% of patients (n = 174) were abstinent at first but not at last urine in 2011, down from 16.9% in 2010 (deteriorating abstinence). We conducted a longitudinal analysis of abstinence and compliance among 17.5% of the 2011 randomized subset. A statistically significant upward trend (p = 2.353 × 10^−8^) of abstinence rates as well as a similar but stronger trend for compliance (p = 2.200 × 10^−16^) was found. Being cognizant of the trend toward drug urine testing being linked to medical necessity eliminating abusive screening, the interpretation of these valuable results requires further intensive investigation.

### Should the US government repeal restrictions on Bup/Nal treatment? [123]

A change is needed to the current US law restricting physicians from prescribing buprenorphine to more than 100 patients for the treatment of opioid use disorder or detoxification. ASAM criteria do not define comprehensive treatment in the current system. The system is fragmented and stigmatized, and ASAM’s call for integrated treatment of addiction will require a significant change. As a result of this commentary, “best practice” should be developed and implemented, and caution should be exercised in lifting the 100-patient limit until the goal has been achieved in a substantial way. As part of the authors’ proposal, the patient limit should be increased to 200 for physicians certified in Addiction Medicine by the ABAM or in Addiction Psychiatry by the American Board of Psychiatry and Neurology, or other medical organizations with a similar certification. A progressive system of rewarding and documenting competence should be followed to lift additional restrictions. A treatment system such as this would integrate treatment, treatment systems, and recovery with prescription medications. A genetic addiction risk assessment should also be incorporated into monitoring emotional blunting, treatment progress, and the initiation of treatment.

### Long term Suboxone^™^ emotional reactivity as measured by automatic detection in speech [124]

In terms of public health and societal issues, illicit drug addiction is among the most critical. Several factors, including the current opioid prescription epidemic, the need for Bup/Nal (Suboxone^®^; SUBX) as an opioid maintenance drug, as well as the growing street diversion of SUBX, motivated the study of long-term SUBX patients’ affective states (“true ground emotionality”). Our aim was to monitor “true” emotionality using emotion-detection in speech in 36 SUBX patients compared to 44 individuals from the general population and 33 members of Alcoholics Anonymous. Patients who are abstinent from heroin, methadone, and opioids have also been examined in less objective studies. Based on these studies, opioid users have abnormal emotional experiences, such as heightened sensitivity to unpleasant stimuli and blunted sensitivity to pleasant ones. To our knowledge, this is the first study to assess long-term Bup/Nal combination (Suboxone^™^) emotionality. Patients with SUBX had significantly flat affect (p = 0.01), and they had less self-awareness of being happy, sad, and anxious than those in general population and Alcoholics Anonymous. These seemingly important results should not be interpreted definitively until we compare opioid-abstinent controls to their emotional reactivity. As a result of these findings, further research is needed to determine which brain regions are responsible for relapse prevention of opioid addiction in SUBX patients.

### Buprenorphine response as a function of neurogenetic polymorphic antecedents: Can dopamine genes affect clinical outcomes in RDS? [125]

There is a plethora of research indicating the successful treatment of opioid dependence with either buprenorphine alone or in combination with naloxone (Suboxone^®^). However, we encourage caution in long-term maintenance with these drugs, albeit lack of any other FDA approved opioid maintenance compound to date. Our concern has been supported by severe withdrawal (even with tapering of the dosage of for example Suboxone^®^ which is 40 times more potent than morphine) from low dose of buprenorphine (alone or with naloxone). In addition, our findings of a long-term flat effect in chronic Suboxone^®^ patients amongst other unwanted side effects including diversion and suicide attempts provides impetus to reconsider long-term utilization. However, it seems prudent to embrace genetic testing to reveal reward circuitry gene polymorphisms especially those related to dopaminergic pathways as well as opioid receptor(s) as a way of improving treatment outcomes. Understanding the interaction of reward circuitry involvement in buprenorphine effects and respective genotypes provides a novel framework to augment a patient’s clinical experience and benefits during opioid replacement therapy.

### Addiction treatment in America: After money or aftercare? [126]

A total of 14,500 clinics and programs provide treatment for addictive behaviors we call “RDS” in America. We propose herein that most of these are not based on scientific evidence, even though they are intended to provide needed help to RDS victims. Following primary treatment, aftercare can include 12-step programs as well as other forms of therapy. While the recovery process is at its most vulnerable, very few programs provide evidence-based treatment approaches. Continuing motivation to use/abuse alcohol or other drugs can be reduced by a hypodopaminergic trait (genetic) and/or state (epigenetic). Drugs approved by the FDA to treat addiction to alcohol, opiates and nicotine may have short-term benefits by blocking dopamine. Instead, we advocate utilizing long-term benefits that induce “dopamine homeostasis”, or “normalcy”. Several holistic methods can be employed to accomplish this, including, but not limited to, dopamine-boosting diets, hyperoxygenation, heavy metal detoxification, exercise, meditation, yoga, and most importantly, nutraceuticals like KB220 variants to balance brain neurotransmitters. The 12-step program, especially aftercare, is something we embrace, but not as a stand-alone modality. Lastly, we provide some scientific explanations as to why resting state functional connectivity (RSFMRI) is so important and how it might be used to treat RDS. As drugs, food, smoking, gambling, and compulsive sexual behavior can reduce rsfMRI, we propose (based on required research) modalities that could restore this impaired crosstalk between various brain areas (e.g., NAc, cingulate gyrus, and hippocampus) should be included in the aftercare program of every treatment program in the US It is estimated that 90% of treatment participants will end up in the “revolving door” if anything less is done.

## Conclusion

In terms of encouraging the field to consider the “RDS Anti- addiction Modeling” consisting of early risk identification with genetic type of assessment similar to the GARS and subsequent induction of dopamine homeostasis utilizing the genetic guided pro-dopamine regulation like KB220, additional research is required.
